# Aesthetic preference in the production of image sequences

**DOI:** 10.3389/fpsyg.2023.1165143

**Published:** 2023-11-30

**Authors:** Ernesto Monroy, Guido Orgs, Noam Sagiv

**Affiliations:** ^1^Department of Psychology, Brunel University London, London, United Kingdom; ^2^Department of Psychology, Universidad del Norte, Barranquilla, Colombia; ^3^Department of Psychology, Goldsmiths, University of London, London, United Kingdom

**Keywords:** empirical aesthetics, method of production, body perception, good continuation, symmetry

## Abstract

**Introduction:**

This research uses the production method to study aesthetic preference for sequences of human body postures. In two experiments, participants produced image sequences based on their aesthetic preferences, while we measured the visual aesthetic features displayed in the compositions.

**Methods:**

In Experiment 1, participants created static image sequences based on their preferences. In Experiment 2, participants sorted images into apparent motion sequences they preferred to view.

**Results:**

In Experiment 1, good continuation of successive bodies and body-like objects was the preferred order. In Experiment 2, participants preferred abstract images with local sequential symmetry and human body postures exhibiting global sequential symmetry.

**Discussion:**

Our findings are compared to those of previous studies that employed the more widely used method of choice. Our experiments propose novel methods and conceptualizations for investigating aesthetic preferences for human body movement and other types of stimulus sequences.

## 1 Introduction

Dance and performing arts are distinct from other traditional fine arts: one of its main aesthetic objects is the artistic representation of the human body moving through time. While traditional visual arts tend to present static aesthetic objects (e.g., paintings), performing arts present moving aesthetic objects, human bodies, and changing actions and positions over time (e.g., dance and performance). Such a distinction in the presentation of aesthetic objects is related to a distinction in the perception of the artistic representation. In this line, the perception of a collection of static stimuli implies independence of each aesthetic judgement evoked in front of each object. However, the perception of a sequence of stimuli implies an interdependence of the aesthetic judgements elicited over time (Khaw and Freedberg, 2018). According to this, the judgements evoked by a collection of paintings are independent because each painting is a work of art in itself. In contrast, aesthetic judgements evoked by a sequence of body postures should directly depend on the context of the postures presented before and after.

In this sense, psychological research on performing arts aesthetics requires divergent studies, different from the traditional ones used for visual arts. To make it possible, our research presents two experiments in which we apply the method of production to the study of human body postures.

In the production method, participants make stimuli applying their individual aesthetic rules, expressing their preferences under different instructions (Westphal-Fitch et al., 2013). An advantage of this approach is that it avoids the researcher's biases toward the creation of stimuli that will be presented to participants (Westphal-Fitch et al., 2013). The production method has been previously used in experiments to examine aesthetic preferences in visual arts (McManus et al., 2010; Westphal-Fitch et al., 2013). However, given the growing interest in experimental aesthetics of performing arts (Orgs et al., 2013; Jola et al., 2014; Kirsch et al., 2015; Christensen et al., 2016), it is worthwhile to extend its application to the study of aesthetic preferences for sequences of body postures that imply movement between postures.

**Figure 1:**
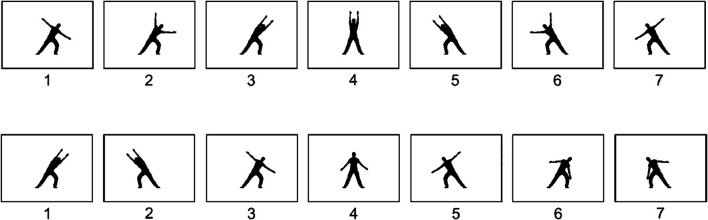
Example of body posture symmetry and continuation. Depicts a digital reproduction of the actual sorting produced by different participants regarding the interesting condition. The upper row illustrates the global symmetry between cards 1 and 7, 2 and 6, and 3 and 5. Moreover, the upper row shows good continuation between cards 1 and 2, 2 and 3, 3 and 4, 4 and 5, 5 and 6, and 6 and 7. The lower row displays an example of local symmetry between the cards 1 and 2 and 6 and 7.

Here, we aim to apply the production method to measure aesthetic features of human movement symmetry and continuation, as they are associated with aesthetic preference, as both features imply higher fluency (Orgs et al., 2016). This means that symmetry and longer continuation require less cognitive effort to process information compared with asymmetry and shorter continuation (Reber et al., 2004; Orgs et al., 2013). An example of sequences varying in movement symmetry and continuation can be seen in Figure 1. Based on existing research in this area with apparent movement sequences (Orgs et al., 2013; Cracco et al., 2022, 2023), we hypothesize that sequences implying fluent movement (in line with the good continuation of visual gestalts) and symmetric sequences will be preferred to non-fluent or asymmetric sequences. Our aim in this study was to replicate these findings using the production method.

Another factor to consider is whether the observed aesthetic object depicts a human body or non-human forms. Some studies have found a correlation between embodied cognition and aesthetic preference for observing familiar movements (Kirsch et al., 2013, 2015). In this case, based on the embodied cognition framework proposed by Glenberg and Kaschak (2002) and Glenberg et al. (2008), as well as the concept of motor resonance introduced by Gallese (2003), it is hypothesized that participants can establish a correlation between their own body movements and the suggested motion of another human body. When observing a sequence of motion implications made with human body images and a sequence produced with abstract shapes and images of objects, participants should relate more to the images that resemble the human body. However, it is less likely to associate them with sequences of abstract shapes and non-human images. Thus, it is hypothesized that the “choreographed” sequences will differ between body postures and inanimate and abstract control stimuli. This is because the latter are not constrained by human movement feasibility. It would be more challenging to link objects or abstract images to one's own body movements.

In summary, it is expected that image sequences with lower resemblance to the human body will exhibit lower symmetry and good continuation. We hypothesize that images with a higher resemblance to the human body will exhibit increased sequential symmetry and good continuation. Participants will produce higher symmetry and continuation when viewing images of the human body, followed by images of corkscrews and scissors. Abstract images will elicit the lowest levels of sequential symmetry and good continuation produced by participants.

In the first experiment, participants produced image sequences either based on liking or based on interestingness. Participants were instructed to generate sequences of still images (or implied motion) that they would *like* to look at and sequences they would find *interesting* to look at. Given that, in everyday life, “like” carries a positive valence and pleasure, the like condition should elicit an appreciation of fluency. Meanwhile, the interesting condition should elicit an appreciation of potentially greater sequence complexity because interestingness is linked to novelty rather than fluency, to something that captures the attention of the observer without necessarily implying pleasure (Earle, 2012). In addition, liking and interest correspond to distinct dimensions of the aesthetic experience. Liking pertains to the evaluative aspect of aesthetic judgement. Interestingness corresponds to the dimension of arousal or intensity, which is related to judgements about stimulus information (Berlyne, 1974; Orgs et al., 2016). Therefore, it is hypothesized that the production of likable image sequences will score higher on sequential symmetry and good continuation than interesting image sequences.

This aligns with the notions of high and low cognitive effort. Therefore, low cognitive effort would be induced in participants when producing sequences they would like to see. High cognitive effort would be required from the participants while producing sequences they deem as interesting to view. We hypothesize that experts are more likely to adopt a high cognitive effort strategy, whereas novices tend to adopt a low cognitive effort strategy when producing image sequences (Orgs et al., 2016). Nevertheless, we will test whether we can induce high or low cognitive effort in novices, through varying instructions related to liking and interestingness.

In regard to the aesthetic output of our participants, we will assess symmetry and continuation. Image symmetry is the replication of a visual pattern, displayed as a mirror image of the original. For instance, observing a pattern with vertical symmetry requires processing less information as it has more redundancy. The left side of the display contains the same information as the right side (Berlyne, 1972; Reber et al., 2004). It is akin to gazing at an object reflected in a mirror.

If we consider different hierarchical levels of a composition (Orgs et al., 2013), local or global symmetry may occur (Figure 1 illustrates an example of this). These hierarchical levels aid in the analysis of the effects of specific features of human movement. For instance, one can assess whether a choreography is favored due to the close connection between adjacent postures (local symmetry), the ample correspondence between past and future postures (global symmetry), or due to the transition between some postures (continuation). Therefore, for measuring continuation, local and global symmetry is useful in assessing their impact on an observer's aesthetic preference.

Local symmetry occurs at the dynamic level (Orgs et al., 2013), in the specific transition between postures, such as the example of an object in front of a mirror mentioned above. This is a mirror symmetry along a vertical axis (Westphal-Fitch et al., 2013). Besides the visual example illustrated in Figure 1, local symmetry can be demonstrated through written examples using letters. Usually, pairs of letters, such as “bd”, “db”, “pq”, and “qp”, exhibit local symmetry with the exception of certain serifs that may vary depending on the font.

Global symmetry exists at the structural level, as demonstrated by Orgs et al. (2013), through the visual balance between distant postures of a sequence of movements. For instance, performing a sequence of movements from left to right by stretching the body to the left, then standing upright, and then finishing by stretching the body to the right. In this case, the first and the last position exemplify global symmetry. We can consider this as a symmetric sequence due to the reflection that creates a balance between leftward and rightward movements. They are the same movements performed at the same pace, with the orientation and timing being the only distinguishing factors, whether the movement is performed to the left or right, and at the start or end of the sequence. In global symmetry, mirrored images are not presented side by side; instead, they are displayed in balanced and more distant positions. In addition to the visual example in Figure 1, a written example of global symmetry can also be demonstrated using letters. The letter sequence “bpdq” exhibits global symmetry with exceptions due to serifs and font variations. In this example, global symmetry is observed between the first and third letters (b and d) and the second and last letters (p and q).

In the context of image sequences, good continuation refers to the smooth transition from one posture to the next one. Continuation in static images is explained by the Gestalt principle of “good continuation.” This principle states that images going in the same direction and in the same sequence or order are typically perceived as part of the same group or as part of the same object (Wertheimer, 1923/1938; Koffka, 1935; Arnheim, 1974; Orgs et al., 2013). In this case, similar sequential postures should be perceived as part of the same sequence. This is relevant as sequences with good continuation are more predictable and easier to process visually. Sequences of contiguous or adjacent body positions flow in a common trajectory implying good continuation (Orgs et al., 2011, 2013). Besides the pictorial example of Figure 1, we could illustrate a good continuation with letters. We can find good continuation in letter strings formed by characters of similar or identical shapes placed side by side. For instance, we can see a good continuation in a string formed by a lowercase “o” and uppercase “O” (e.g., oOoO). Moreover, a dinkus, a string of asterisks used in typography as a section heading can show good continuation (e.g., ^*^^*^^*^^*^).

Finally, Experiment 2 is a follow-up study that compared the production of liked and disliked digital animations that displayed apparent motion, using abstract and human body images. More details about this study of the production of animations are presented in the introduction section of Experiment 2.

## 2 Experiment 1

In Experiment 1, we asked participants to produce sequences of static images that they would like to see or that they would find interesting to see. After they produced the sequences, we measured their continuation and symmetry to determine whether participants liked or were interested in symmetric sequences and sequences with good continuation. Both experiments were approved by the Ethics Committee of Brunel University London.

### 2.1 Methods

#### 2.1.1 Participants

Participants can be characterized as non-experts in the field of arts. The sample comprised of 28 Brunel University students (25 women)with 25 being undergraduates recruited through Brunel University London's online participant pool system and the remaining four contacted through referral sampling. The average age of the participants was 19 years old (age range = 18–27 years). A total of 26 first-year Psychology students were given credits for their participation, with 17 of them holding British nationality while the rest came from Europe, Asia, and Africa. Seven of the participants had no prior artistic education. None of the participants disclosed professional art training. Seven received art classes during their childhood, eight at a recreational or exercise level, and six at a vocational or teaching level. The training focussed on three artistic domains: performing arts (11), visual arts (8), and music (2). The average length of time since participants last took art classes was 4.38 years ago (*M* = 4.38, *SD* = 4.17). The average number of years that participants took art classes was 3.64 (*M* = 3.64, *SD* = 2.65). On average, participants made 2.89 annual visits to museums (*M* = 2.89, *SD* = 4.54). Finally, participants watched performances an average of 4.6 times per year (*M* = 4.6, *SD* = 8.63). The sample size calculated using G^*^Power (Faul et al., 2007) was determined to be 16 participants for a medium effect size and a statistical power of 0.80.

#### 2.1.2 Procedure

An adapted version of the card sorting technique (Rugg and McGeorge, 1997; Nurmuliani et al., 2004) was utilized for the experiment. Based on their preferences, participants arranged printed cards into image sequences that they liked and image sequences that they found interesting, as instructed by the researcher. The participants were made aware that there were no correct or incorrect sequences and that they should arrange the order of the images purely based on their own aesthetic criteria.

**Figure 2:**
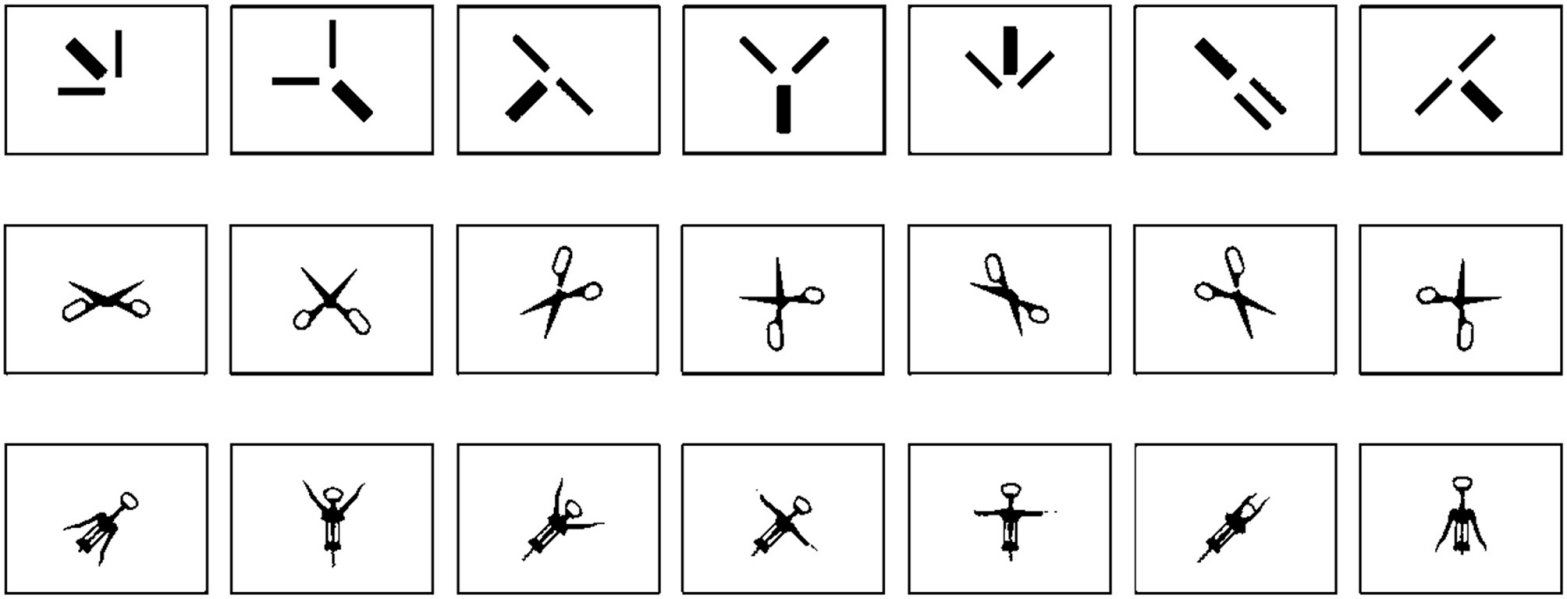
Examples of abstract, scissors, and corkscrew cards. This figure demonstrates a digital reproduction of the actual sorting completed by various participants. The upper row displays abstract images (created for the liked condition), the middle row exhibits images of scissors (created for the interesting condition), and the lower row depicts images of corkscrews (created for the interesting condition).

The procedure was conducted with various sets of images for replication. There were four decks of cards: abstract images, scissor images, corkscrew images, and body postures. A printable file containing the images utilized for Experiment 1 can be accessed via Supplementary material. The images were based on those utilized by Orgs et al. (2013). Each deck contained 12 cards, each depicting a distinct position of the same object (see Figures 1, 2). The printed cards were sorted on a desk at a viewing distance of 40 cm. The length and width of each card were 10.04 cm and 7.38 cm, respectively. If arranged in a specific order, the cards could indicate a sequence of movement for a particular object. For example, the motion implied in opening or closing a pair of scissors. Once sorted, the cards would be arranged in a simplified version of a storyboard.

Participants were allotted a maximum of 5 min per sorting task but were encouraged to use less time if necessary. This duration was determined after conducting pilot studies with the research team and was confirmed to be appropriate during both experiments. The average duration for sorting in Experiment 1 was 95.55 s. In Experiment 2, it took participants an average of 93.34 s to produce each digital animation. After sorting each set of cards, participants informed the researcher to record the duration of the sorting process using a stopwatch. Then, the researcher took a photograph of the results before proceeding with the next set of cards.

Participants were instructed to follow these steps:

1. See a set of 12 cards.
2. Choose seven cards.
3. Sort the selected images in a set of seven cards.
4. Each set must contain seven cards.
5. The set of the selected cards must be arranged horizontally, from left to right.

Participants were provided with an informational document and a verbal explanation of the experiment prior to signing a consent form. Then, the participants reviewed the instruction page and inquired for clarifications if necessary. To validate the information, the experimenter provided a brief explanation of the procedure. Subsequently, the cards were handed out and the participant began the sorting procedure. Then, the experimenter shuffled the cards and repeated the process for the “like” or “interesting” condition with each randomized deck of cards. After the sorting procedure, participants completed a short questionnaire and were debriefed.

#### 2.1.3 Measures

The measures were global symmetry, local symmetry, and continuation. *Global symmetry* refers to mirrored images in distant pairs of cards: the first with the last, the second with the penultimate, and the third with the antepenultimate [Phrase Structure Grammar in Bahlmann et al. (2006)]. It was scored on a scale of 0 to 3 based on the presence of global symmetry, for which each pair of symmetrical cards received 1 point. *Local symmetry* consisted of mirrored images closed together one next to each other [as defined in the finite state grammar by Bahlmann et al. (2006)]. It was scored on a scale of 0 to 3. One point was awarded to each pair of cards exhibiting local symmetry. *Continuation* examines the similarity between adjacent images and the implied motion transition between them, in accordance with Orgs et al. (2013). It was rated on a scale of 0 to 6 based on whether each pair of cards had a “good” continuation, which scored 1 point. As previously noted in the Introduction section, good continuation is the arrangement of adjoining images that imply a smooth transition between one image and the next one.

The scoring of symmetry and continuation was performed by sorting cropped and printed photographs manually. Each sorting photograph was compared to a reference template with the greatest symmetry and continuation. The scoring procedure was performed twice to ensure accuracy. For instance, the upper row of Figure 1 exhibits that we can score three points for global symmetry: images 1 and 7 (1 point), 2 and 6 (1 point), and 3 and 5 (1 point). In the lower row of Figure 1, there is one point for global symmetry: images 3 and 5. For Figure 1 (lower row), local symmetry scores two points: images 1 and 2 (1 point) and images 6 and 7 (1 point). In Figure 1 (upper row), the continuation score consists of six points: images 1 and 2 (1 point), 2 and 3 (1 point), 3 and 4 (1 point), 4 and 5 (1 point), 5 and 6 (1 point), and 6 and 7 (1 point).

Finally, we administered a *background questionnaire*. The questionnaire utilized for this analysis of performing arts was adapted from The Watching Dance Project (Reason and Reynolds, 2010). It included inquiries about demographic data, artistic history, standards for card sorting, and preferred sets of cards. The questionnaire contained queries on age, gender, and nationality. Questions about artistic background were as follows: Have you ever taken arts, dance, or performing arts classes? How long ago did you take classes? How many years have you studied art, dance, or performing arts? How would you best describe your level of training? What style/form of dance (or arts/performing arts) are you trained in? How many times per year do you visit art museums or galleries? How many times per year do you watch dance performances or performing arts? What were your criteria for arranging the cards?

As the aesthetic appreciation of observers may be influenced by their level of artistic expertise (Hekkert and van Wieringen, 1996; Furnham and Walker, 2001; Illes, 2008; Uusitalo et al., 2009; Pihko et al., 2011), we collected data on the educational and artistic background of participants to determine whether they were novices in artistic domains.

#### 2.1.4 Research design

The research design comprised four sets of images and two counterbalanced sorting procedures per set: like to see and interesting to see. As a result, each participant completed a total of eight sortings. The order of the image conditions was fixed because they were presented from the most abstract to the most concrete reference to the human body. All images resembled a human body in terms of head, trunk, and limbs in the following order, from abstract to concrete: (1) abstract images, (2) scissors, (3) corkscrew, and (4) body postures. In this manner, the abstract images served as a control stimulus by presenting them at the start of the procedure, thereby limiting potential associations to the human body.

The order of the instructions, “images that you would like to see” and “images that you would consider interesting to see” was randomly assigned to counterbalance the sorting under the “like” condition before the “interesting” condition, or vice versa.

### 2.2 Results (Experiment 1)

#### 2.2.1 Comparison between images: abstract, scissors, corkscrew, and body postures

In order to test whether participants produced higher symmetry and continuation when sorting static sequences using different types of images (abstract, objects, and human body), we compared the numerical scores across images. Non-significant results (*p* > 0.05) are reported in detail in Supplementary material.

**Figure 3:**
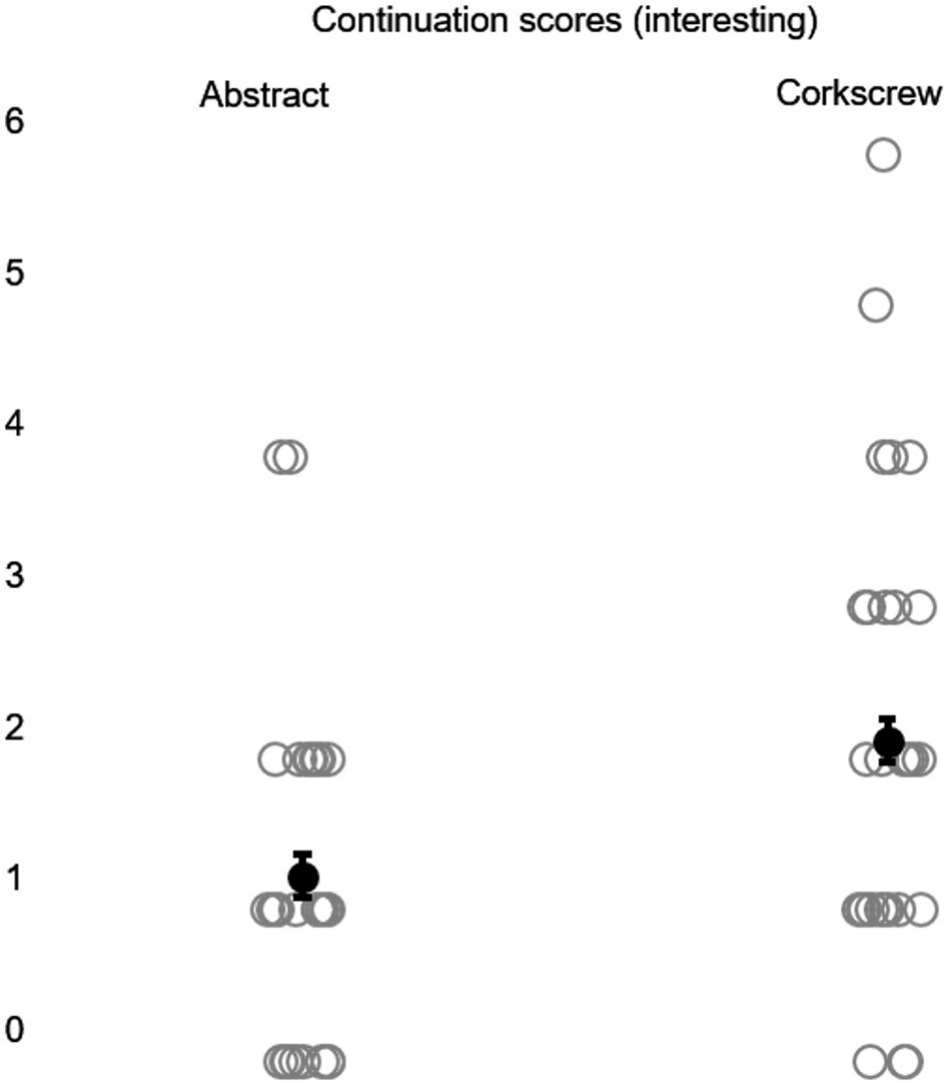
Continuation scores produced with abstract and corkscrew images, under the indication of interestingness, in the card sorting experiment (Experiment 1). Gray circles represent data points; black dots represent mean scores. Error bars represent the standard error of the mean (SEM).

Friedman's ANOVA revealed a significant difference in the “interesting” condition with respect to continuation [χ^2^_(3)_ = 10.32; *p* < 0.05], indicating different image sets had a significant difference. A Wilcoxon signed-rank *post-hoc* test, using a Bonferroni correction (level of significance 0.0083), indicated that corkscrew continuation was significantly higher than abstract continuation (T = 34; r = −0.36) (see Figure 3). Friedman's ANOVA revealed no significant difference among abstract images, scissors, corkscrews, and postures concerning global symmetry, local symmetry, and continuation under the “like” condition. The same result was obtained for the “interesting” condition regarding global symmetry and local symmetry.

#### 2.2.2 Comparison between indications: sequences that you would like to see vs. images that you would consider interesting to see

To test whether participants produced sequences with higher global symmetry and good continuation for the like condition, we compared the scores between like and interesting conditions on each of the aesthetic features. None of the comparisons resulted in a significant difference. Non-significant results are reported in detail in Supplementary material.

#### 2.2.3 Self-report measures

After the sorting task, participants completed a printed questionnaire by hand. One question on the questionnaire asked, “What were your criteria for arranging the cards?” To analyse these responses, all answers were transcribed into a table. In order to explore whether participants reported considering the implied motion, we categorized the data into two groups based on their self-reported responses. Considering the content of the answers, we categorized them into two groups: those that explicitly mentioned the notion of movement and those that did not. If the response mentioned words such as action, movement, motion, sequence, or order, the response was assigned to the category of movement. If the response did not mention any of those words, it was assigned to the category without movement. We wrote the category in front of each transcribed answer.

An example of an answer categorized into the group that considered implied motion was as follows: “At first it was a little random, I didn't really think about it. But then, I saw the pattern and movement that could be created by placing them in different places. Were more everyday poses (like). Were the more unusual poses, especially with the last set (interesting)” [SIC]. An answer categorized into the group that did not consider implied motion was: “The shapes which went off to the left I found least interesting than the central ones or ones to the right; the symbols were organized to things I associated it with such as flowers. I liked the ones that went more to the right. Larger, weird shapes were more interesting.”

Following this categorization, we found that nine participants produced the sequences based on implied movement while the aesthetic production of 19 participants did not rely on implied movement.

Another question on the printed questionnaire was “What was your favorite set of cards?” According to the responses of the participants, the favorite set of cards was postures (11), followed by corkscrew (10), scissors (5), and abstract (2).

### 2.4 Discussion (Experiment 1)

The objective of Experiment 1 was to apply the production method to better understand the aesthetics of image sequences of body and non-body stimuli and to test whether ordering such sequences would take into account the implied movement between successive images of a sequence. The study compared sequences based on interestingness and liking and for abstract shapes, objects, and body postures. The analysis was complemented with self-reported information provided in the background questionnaire.

When comparing the conditions of “like” vs. “interesting,” it was expected to find higher continuation and global symmetry for the “like” condition and lower scores for the “interesting” condition. This prediction was not supported as there were no significant differences between like and interesting.

The lack of significant results in this analysis indicates the similarity between like and interesting for non-experts. In theory, these concepts are distinct, yet in practice, we found that they do not differ enough to produce a significant impact on the order of image sequences. These results suggest that for our participants “like” and “interesting” were not sufficiently separate concepts, at least when applied in the context of the method of production.

The results demonstrated a significant difference in the continuation production of sequences with corkscrew images vs. abstract images when generated under the instruction of interestingness. The abstract images' low continuation suggests that this serves as an appropriate control condition. It can be interpreted as a low familiarity stimulus that does not have a clear reference to the human body or to a physical object and its physical rules. This can result in participants finding it difficult to produce a clear aesthetic pattern in terms of continuation. This discovery may suggest the impact of familiar and meaningful object categories on aesthetic preference when generating visual sequences.

It was hypothesized that global symmetry and good continuation would be liked and that local symmetry and low continuation would be interesting. As we applied the production method, symmetry and continuation are our dependent variables. Participants produced interesting or liked sequences, and then, we measured whether these sequences were characterized by more or less symmetry and continuation. As 9 out of 28 participants reported considering implied motion when arranging the cards, future studies could explicitly instruct participants to consider implied motion when producing the sequences to keep this rate constant across participants. Another alternative could be the presentation of apparent moving images for the sorting procedure (this was implemented in Experiment 2). In Experiment 1, we did not present any of those scenarios to test the spontaneous response from novice participants. In Experiment 2, we presented animations to test such questions.

In Experiment 1, participants were asked to sort static images they would like to see and that they would consider interesting to see. The results indicated that most of the participants did not consider implied motion during their card arrangement. Moreover, it seems that participants did not distinguish between liking and interestingness judgements. Moreover, participants produced similar levels of symmetry and continuation across images, except for the corkscrew continuation when compared to continuation of abstract images (regarding the judgement of interestingness). Nevertheless, based on self-reports, participants favored corkscrews and human body images as their top choices. Abstract images were the least preferred.

Experiment 2 is a follow-up experiment that addresses the shortcomings of Experiment 1. In Experiment 2, participants were asked to produce abstract and human body animations that they would like to see and those they would not. Thus, participants saw apparent movement (digital animations), generated animations assuming a low cognitive effort strategy (like-dislike), and produced animations of human body and abstract images.

Considering that postures and corkscrews were the preferred set of cards, while abstract was the least favored, it is possible to utilize these sets of images for follow-up experiments due to their contradictory outcomes regarding movement preference and image preference. Abstract images should be utilized in follow-up experiments because they are the least favored in terms of image liking and serve as an appropriate control stimulus in the task of implied motion production. Posture images also should be utilized as an appropriate experimental stimulus for generating motion sequences directly linked to the human body.

Finally, Experiment 1 included a group that used implied motion as a sorting criterion and another group that did not. This highlights the importance of controlling stimuli presentation to prevent varying interpretations of the instructions regarding motion. Experiment 2 addressed this issue by explicitly presenting digital animations to participants, instead of displaying static images.

## 3 Experiment 2

Experiment 2 further explores the idea that the aesthetics of image sequences are related to the movement that is implied by the sequence. This is accomplished by combining the production method with an apparent motion paradigm as opposed to using paper cards to produce image sequences. These aesthetic preferences were studied using the production of animated sequences or GIFs (Graphic Interchange Format files with the .gif extension). Using the same images as in Experiment 1, participants were asked to generate animated GIFs based on their own aesthetic criteria.

Apart from generating GIFs instead of static image sequences, Experiment 2 largely resembles Experiment 1 with a few exceptions. First, Experiment 1 shows no significant difference between liking judgements and interestingness judgements; therefore, we limited Experiment 2 to generating sequences based on liking. Participants were asked to produce animations they liked and animations they disliked. It is expected that participants will produce animations with higher sequential symmetry and good continuation for liked sequences than for disliked sequences. Importantly, participants will see image sequences animated on a computer screen. In Experiment 1, only a minority of participants mentioned motion as an aesthetic criterion for generating aesthetically pleasing image sequences using static images. In Experiment 2, all participants viewed animations of their image sequences.

As we aimed to explore aesthetic preferences for human motion, Experiment 2 employed human body postures. As a control stimulus, abstract images were employed as they are distant from the human body resemblance. Here, we expect higher global symmetry and continuation produced with body images, for the liking condition.

### 3.1 Methods

#### 3.1.1 Participants

The final sample consisted of 28 art domain non-experts (24 women), with 27 being Brunel University students and one being referred to as an external volunteer. Participants were recruited through Brunel University London's online participant pool system and referral sampling. Twenty-four first-year Psychology students received credits. One participant received credits but was excluded from the final sample because it was apparent that the student was not fully engaged with the procedure. The mean age was 20 years (age range = 18–32 years). Nationalities were either British or British/dual (22), from European countries (4), Indian (1), or Congolese (1). Eight participants had no prior artistic education. None of the participants had received training at a professional level, seven had received art classes during their childhood, while 10 had taken them at a recreational level and five at a vocational/teaching level. The areas of artistic training included performing arts (13), visual arts (4), and music (1). The average time since participants last took art classes was 5.47 years (*M* = 5.47, *SD* = 3.80). The average number of years that participants took art classes was 3.14 (*M* = 3.14, *SD* = 3.97). On average, participants visited museums 1.81 times per year (*M* = 1.81, *SD* = 1.86). Participants also attended performances an average of 3.13 times per year (*M* = 3.13, *SD* = 2.90). The educational background of participants consisted of Psychology (26 participants), Law (1 participant), and Marketing (1 participant). The educational level was undergraduate for 24 participants and graduate for four participants. The sample size was calculated using G^*^Power (Faul et al., 2007) for a medium effect size, and a statistical power of 0.80, which determined a sample size of 24 participants.

#### 3.1.2 Procedure

A digital adaptation of the card sorting method was applied. Participants created digital animations with apparent movement (GIFs) by sorting digital image sets based on their preferences and sets they found unappealing, following the researcher's instructions. It was clarified that there were no correct or incorrect answers and that it depended on individual criteria. Participants created the animations using the software PhotoScape. The animation speed remained constant at 150 ms as previous studies have indicated that participants find animated sequences most appealing when presented at this rate (Orgs et al., 2013). The onscreen display of PhotoScape maintained consistent proportions, enabling participants to view (1) all the randomized images on the left side of the screen, (2) the images they were sorting by dragging on the top of the screen, and (3) the animation itself positioned at the center of the monitor. Twin mouse/keyboard setups were installed so that the participant sat in front of a computer screen with a mouse and a keyboard, and the researcher was seated at an adjacent desk with an additional mouse/keyboard to load the images before each sorting task and to save the screenshots and animations after each production task.

The procedure was conducted with sets of images: abstract images or a human body back view (images can be found in Experiment 2 in the Supplementary material). The abstract and posture images that were utilized in Experiment 2 were the same as those used in the sequence production experiment (Experiment 1). Each group of images consisted of 12 pictures, with each picture depicting the same object in various positions. The digital images were projected on a 15-inch monitor, viewed from 50 cm. The dimension of the digital image was 20.74 cm in length and 15.24 cm in width. Participants were given a maximum of 5 min to create their animations, but they were encouraged to use less time if necessary. Once each group of images was sorted, participants notified the researcher in order to register the sorting time with a stopwatch and then to take a screenshot of the results before moving on to the next set of images.

Participants were first provided with an information sheet followed by a verbal explanation of the experiment, prior to signing a consent form. Subsequently, they were instructed to read an instruction page and to ask any questions they may have had. To validate the information, the experimenter provided a brief explanation of the procedure. Later, the participant started the sorting procedure upon being presented with randomized images.

A.gif file was saved for each animation. This process was then repeated for the randomized “like” or “dislike” condition for each set of images. Following the sorting procedure, participants answered a brief questionnaire and were debriefed.

#### 3.1.3 Measures

Measures for Experiment 2 were identical to those in Experiment 1. These included assessments of global and local symmetry which were scored on a scale of 0 to 4 as the extremes were also taken into account, and good continuation of movement which was scored on a scale of 0 to 7 for the same. The background questionnaire was identical, except that this time it inquired if participants perceived a distinction between like and dislike. The scoring of sequential symmetry and movement continuation was executed in the same way as conducted in Experiment 1.

### 3.2 Results (Experiment 2)

#### 3.2.1 Making liked and disliked sequences

To determine whether participants produced higher global symmetry and higher continuation for their liking judgements and whether they produced higher local symmetry for their disliking judgements, we analyzed symmetry and continuation scores produced for both liked and disliked sequences. Non-significant results are reported in detail in Supplementary Material.

**Figure 4:**
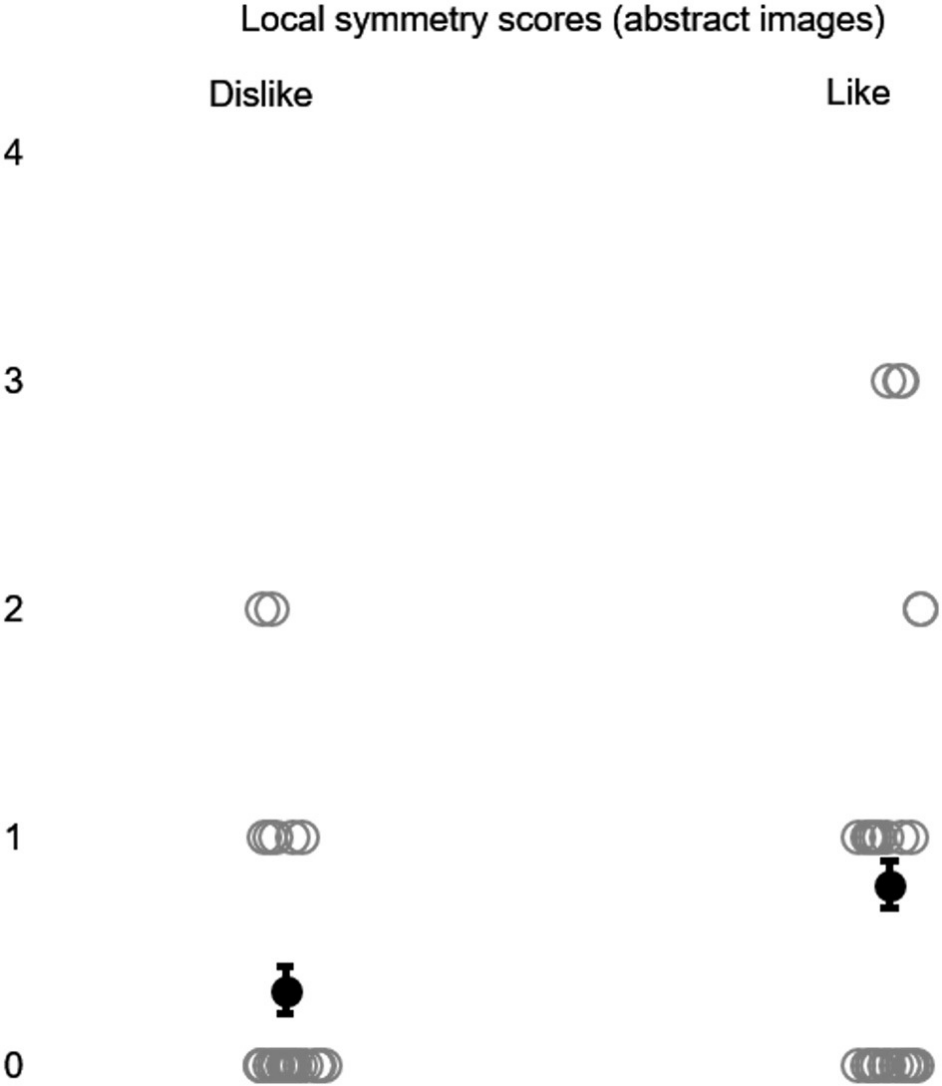
Local symmetry scores were generated using abstract images in the production of the animations (Experiment 2) as indicated by participants' preferences for liking or disliking. Gray circles represent data points; black dots indicate mean scores. Error bars represent SEM.

**Figure 5:**
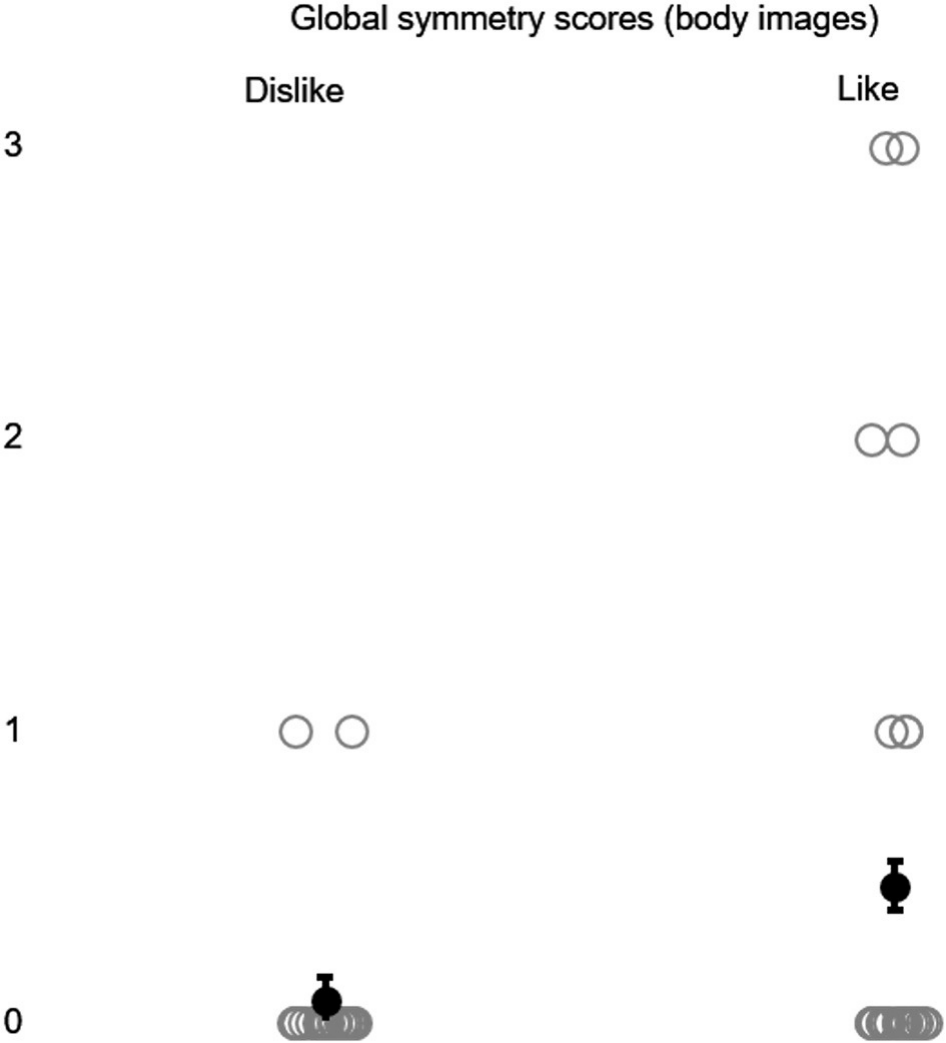
Global symmetry scores were generated with body images in the animations (Experiment 2) and were categorized under the indications of liking and disliking. Gray circles represent data points, while black dots represent mean scores. Error bars represent SEM.

A Wilcoxon signed-rank test demonstrated a significantly higher local symmetry with abstract images for liking (*Mdn* = 0.50) than for disliking (*Mdn* = 0.00), *z* = −2.14, *p* < 0.05, *r* = −0.29 (see Figure 4), and *a* significantly higher global symmetry with postures for liking (*Mdn* = 0.00) than for dislike (*Mdn* = 0.00), *z* = −2.21, *p* < 0.05, *r* = −0.29 (see Figure 5).

There were no other significant comparisons (all *p* > 0.05).

#### 3.2.2 Making abstract and body posture sequences

In order to test whether participants produced higher sequential symmetry and good continuation scores with body postures than with abstract images, we compared the symmetry and continuation scores produced for abstract and body sequences. None of these comparisons revealed significant differences. In the Supplementary material, we report non-significant results in detail.

#### 3.2.3 Self-reported measures

All participants reported finding a difference between the animations they created for liking judgements and those they created for disliking judgements. According to the descriptions provided, the criteria for producing liked animations were characterized under the following non-mutually exclusive categories (cases may appear in more than one category): order/continuation (19), fluency (11), symmetry (5), natural/real (5), positive valence (3), variety (2), high arousal (1), synchrony (1), big (1), and interestingness (1). The criteria for producing disliked animations were inclusively categorized as (cases may appear in more than one category): random/disorder (19), abrupt/disfluent (5), simple (3), asymmetry/unbalanced (2), small (1), vigorous (1), and negative valence (1). Participants reported that their favorite animations were those produced using body postures for liking judgements (20), followed by animations producing abstract images for liking judgements (6), animations produced with body postures for both liking judgements and disliking judgements (1), and no favorite animation (1).

### 3.3 Discussion (Experiment 2)

In Experiment 2, it was hypothesized that participants would produce higher levels of global symmetry and higher continuation for liking judgements' animations and higher levels of local symmetry for the disliking judgements' animations. In addition, it was anticipated that participants would produce animations with higher symmetry and continuation levels when using body postures than abstract images.

In Experiment 2, participants produced animations that exhibit higher global symmetry for liking judgements than for disliking judgements, when sorting human body images. This finding can be interpreted as non-experts having a preference for aesthetic objects with higher global symmetry. Such an aesthetic preference for producing symmetrical sequences aligns with prior studies that have employed the method of choice to study the aesthetic perception of image sequences (Orgs et al., 2013; Khaw and Freedberg, 2018; Cracco et al., 2022, 2023). One potential explanation is that higher global symmetry can be interpreted as higher fluency as symmetric stimuli are easier to process (Orgs et al., 2016).

In addition, these findings align with other conceptual frameworks that prioritize simplicity and familiarity over complexity and novelty, such as repeated exposure in the mere exposure effect (Zajonc, 1968), figure-ground contrast in Gestalt theories (Arnheim, 1974), and prototypicality (Martindale and Moore, 1988). In this sense, aesthetic features, such as symmetry, repeated exposure, figure-ground contrast, and prototypicality, enhance fluency. As a result, in turn, aesthetic preference increases (Reber et al., 2004). From the perspective of processing fluency, these frameworks share the notion that individuals have a preference for aesthetic objects that are simple, familiar, and typical as they are more effortless to process.

Such preference for symmetry is supported by the descriptions provided by participants, with most of them mentioning criteria congruent with order, symmetry, and fluency. According to Khaw and Freedberg (2018), it can be argued that these theoretical approaches share the notion of time and previous experiences. In this manner, a symmetric sequence displays various images that are continuously experienced by the observer.

As the transition from one image to the next mirrored image is considered a bad continuation, we hypothesized higher local symmetry in the disliked animations. However, we found higher local symmetry in the liked animations produced with abstract images. This suggests that participants do not apply compositional rules of local symmetry to produce disliked animations while perceiving apparent motion, at least with abstract images.

## 4 General discussion

We employed the method of production to explore the empirical aesthetics of human motion. Experiment 1 used static pictures to assess the aesthetic criteria for producing image sequences, and Experiment 2 extended this approach by showing participants animations of the image sequences they produced.

In Experiment 1, participants produced similar levels of symmetry and continuation for judgements of liking and interestingness. In addition, participants produced similar levels of symmetry and continuation among the majority of the images (abstract, scissors, corkscrews, and human body). We found significant differences in the judgement of interestingness between the continuation produced with images of corkscrews, which was higher than the continuation produced with abstract images. Interestingly a recent EEG study, comparing body and corkscrew sequences using the same stimuli, showed increased neural amplitudes for corkscrew sequences than for body sequences (Cracco et al., 2023). Higher interestingness and greater neural amplitudes might well reflect an uncanny valley effect. The corkscrew's movement resembles the mechanics of human movement, yet is at the same time clearly identifiable as an inanimate object.

In Experiment 2, participants produced sequences with similar levels of sequential symmetry and good continuation between images (abstract and human body). However, notable distinctions emerged. Participants produced animations with higher local symmetry for liking judgements than disliking judgements, when sorting abstract images. Additionally, participants produced animations with higher global symmetry for liking judgements than for disliking judgements, when sorting human body images.

Regarding Experiment 1, participants utilized good continuation as a compositional rule when dealing with corkscrew sequences as compared to abstract sequences, based on interestingness indications. Despite a limited number of participants considering implied motion while arranging the cards, this was evident. Additionally, it can be inferred that participants did not distinguish between interestingness and liking. The card sorting technique was an initial step in applying the method of production to the aesthetics of image sequences.

A limitation of Experiment 1 is that participants were unable to change cards during the procedure, resulting in more restrictions for producing a sequence. They had to choose the cards and sort them without any flexibility. In contrast, Experiment 2 addressed this limitation by allowing participants to see the animation in the process with the option to rearrange the images as necessary, increasing their flexibility in producing the animation.

In reference to Experiment 2, there were no significant differences observed between body and abstract images. Results from Experiment 1 indicate that when the stimuli are static, participants need an explicit reference to everyday objects to take into account the implied motion as an aesthetic feature of the sequence. When participants perceive apparent motion, non-experts produce sequentially symmetric sequences, regardless of whether the visual stimulus is abstract or whether it explicitly depicts the human body. In other words, when sequences are animated, the fluency of apparent movement becomes an increasingly important aesthetic criterion for generating image sequences that do not depend on what kind of images are being animated (human or not human).

One possible explanation for the lack of stimulus category effects is that, in the method of production, our participants engaged with these stimuli for a relatively extended period during production instead of just a few hundred milliseconds. This shows that both abstract and human body sequences may rely on the same compositional rules (good continuation and sequential symmetry) to produce aesthetically pleasant apparent motion. In contrast, perceptual studies have uncovered variations in the visual processing of biological and non-biological motions (Neri et al., 1998; Grossman and Blake, 2002; Poom and Olsson, 2002; Hiris, 2007; Pyles et al., 2007; Simion et al., 2008; Orgs et al., 2011). This may be attributed to methodological variations. Such differences can be found in stimuli duration and the nature of the task. Perceptual experiments typically use brief stimuli durations in periods of seconds or milliseconds, and the task requires detection or recognition of stimuli presented by the researcher. On the other hand, the method of production employs lengthier stimuli displays, lasting minutes, and the task requires the generation of sequences by the participant.

In Experiment 1, we found a significant result when comparing figurative sequences to abstract images. In this case, participants produced a better continuation with figurative stimuli (corkscrews) than with abstract images. This suggests that fluent implied movement as an aesthetic feature is more important for meaningful objects than for meaningless shapes. Here, we found no significant differences between different figurative images (i.e., scissors, corkscrews, or human bodies).

We observed no significant difference between liked and interesting image sequences. Perhaps, liking and interestingness were not sufficiently different criteria for our non-expert participants. In the method of choice, it has been argued that novices' aesthetic preference may reflect a low cognitive effort strategy, which is hypothesized to be relevant for liking judgements, but not for interestingness judgements (Orgs et al., 2016).

Considering this, in the method of production, if novices are asked to produce aesthetically pleasing image sequences, it should be assumed they would produce them based on liking judgements. In turn, if participants are asked to produce interesting aesthetic objects, it is expected that novices assume a high cognitive effort strategy. However, our results indicate otherwise. Probably, participants' aesthetic production was following a low cognitive effort strategy for both judgements as they were producing liked sequences in both scenarios. We must note that in Experiment 1, the notion of movement was not mentioned in the instructions and the majority of participants did not consider implied movement when arranging the cards. Nonetheless, participants produced higher continuation with corkscrews and lower continuation with abstract shapes. Based on this, it can be inferred that good continuation is more important for the production of figurative image sequences than for the production of abstract image sequences.

In Experiment 2, participants viewed apparent motion and produced animations related to liking and disliking judgements. In the second experiment, no differences were found when comparing the human body against abstract images. However, we discovered higher local symmetry in abstract sequences for liking judgements than disliking judgements. Additionally, global symmetry produced with human body images was higher for liked sequences than for disliked sequences.

This means that, in Experiment 2, participants based their production on differences in global symmetry for liking and disliking when arranging human body images. In contrast, participants preferred abstract animations with higher local symmetry. As discussed in the second experiment, animations with local symmetry imply less good continuation, as transitioning from the original image to the mirrored version of the stimuli leads to an abrupt transition. These findings are therefore consistent with the idea that implied or apparent movement matters more for the aesthetics of figurative sequences than for abstract sequences. In other words, when producing animations, participants prefer abstract sequences with lower good continuation and human body sequences with higher good continuation.

Overall, participants reported a preference for human body and corkscrew sequences over abstract sequences. This is evidenced through the open question regarding favorite sorting and animations, where the majority of participants stated that they preferred body postures and corkscrews. Such results are consistent with prior research indicating that novices prefer representative over abstract art (Hekkert and van Wieringen, 1996; Furnham and Walker, 2001; Illes, 2008; Uusitalo et al., 2009; Pihko et al., 2011). Our two experiments which applied the method of production, extend these findings to sequences with static images and sequences with apparent motion. Animations that more accurately represent actual human movements are preferred to sequences that show the movement of abstract shapes.

One way to understand this is through the embodied cognition framework (Glenberg and Kaschak, 2002; Glenberg et al., 2008) that is connected to the aesthetic perception of human movement (Calvo-Merino et al., 2008; Daprati et al., 2009; Cross et al., 2011; Kirsch et al., 2013, 2015). Viewing human or human-like figures might facilitate taking into account implied or apparent motion when generating the image sequences.

Participants applied similar compositional rules to sequences of abstract and figurative images. However, they prefer figurative sequences. Such application of compositional rules to various stimulus categories is consistent with prior research demonstrating that participants can implicitly follow grammar rules while observing sequences produced with different types of stimuli, including letters, static body images (Norman and Price, 2012), apparent biological motion (Orgs et al., 2013), and dance (Charnavel, 2019).

In other words, the aesthetic movement executed by a concrete performer will be preferred to a similar aesthetic motion executed by an abstract object. Based on the findings of these two experiments, it is plausible to propose that aesthetic preferences for human movement are influenced by both the fluency of perceived motion and the visual embodied representation of the object that is performing the action. As observed in the present experiments, this serves as a foundation to apply the method of production for exploring aesthetic preferences in human movement through the card sorting technique and the production of animations.

## Data availability statement

The raw data supporting the conclusions of this article will be made available by the authors, without undue reservation.

## Ethics statement

The studies involving humans were approved by Brunel University London Ethics Committee. The studies were conducted in accordance with the local legislation and institutional requirements. The participants provided their written informed consent to participate in this study.

## Author contributions

EM contributed to conceptualization, writing, editing, data collection, and analysis. GO and NS contributed to conceptualization and writing and editing. All authors contributed to the article and approved the submitted version.
