# Supplementary material for: Aesthetic preference in the production of image sequences
Source: Front Psychol. 2023 Nov 30;14:1165143. doi: 10.3389/fpsyg.2023.1165143 (PMC10720618; doi:10.3389/fpsyg.2023.1165143)
Supplement: Supplementary file 1 [file Table_1.DOCX]

**Aesthetic preference in the production of image sequences**

**Non-significant results (experiment 1)**

**2.2.1 Comparison across Images: Abstract, Scissors, Corkscrew, and Body Postures**

A Friedman’s ANOVA did not show a significant difference among abstract images, scissors, corkscrew and postures regarding global symmetry (χ^2^ (3) = 1.77, *p* > .05), local symmetry (χ^2^ (3) = 4.69, *p* > .05) and continuation (χ^2^ (3) = 6.77, *p* > .05) under the “like” condition. The same happens for the “interesting” condition regarding global symmetry (χ^2^ (3) = 1.05, *p* > .05) and local symmetry (χ^2^ (3) = 2.73, *p* > .05).

**2.2.2 Comparison between Indications: Sequences that you would Like to See Vs Images that you would Consider Interesting to See**

A Wilcoxon signed-rank test compared the scores between like and interesting on each of the aesthetic features. None of the following comparisons showed a significant difference:

- Liked abstract global symmetry (*Mdn* = 0) compared to interesting abstract global symmetry (*Mdn* = 0), T = 33, *p* > 0.05.
- Liked abstract local symmetry (*Mdn* = 0) compared to interesting abstract local symmetry (*Mdn* = 0), T = 20.50, *p* > 0.05.
- Liked abstract continuation (*Mdn* = 1) compared to interesting abstract continuation (*Mdn* = 1), T = 103.50, *p* > 0.05.
- Liked scissors global symmetry (*Mdn* = 0) compared to interesting scissors global symmetry (*Mdn* = 0), T = 5, *p* > 0.05.
- Liked scissors local symmetry (*Mdn* = 0) compared to interesting scissors local symmetry (*Mdn* = 0), T = 16, *p* > 0.05.
- Liked scissors continuation (*Mdn* = *2*) compared to interesting scissors continuation (*Mdn* = 1), T = 65.50, *p* > 0.05.
- Liked corkscrew global symmetry (*Mdn* = 0) compared to interesting corkscrew global symmetry (*Mdn* = 0), T = 5, *p* > 0.05.
- Liked corkscrew local symmetry (*Mdn* = 0) compared to interesting corkscrew local symmetry (*Mdn* = 0), T = 16, *p* > 0.05.
- Liked corkscrew continuation (*Mdn* = 1) compared to interesting corkscrew continuation (*Mdn* = 2), T = 59.50, *p* > 0.05.
- Liked posture global symmetry (*Mdn* = 0) compared to interesting corkscrew posture symmetry (*Mdn* = 0), T = 20.50, *p* > 0.05.
- Liked posture local symmetry (*Mdn* = 0) compared to interesting posture local symmetry (*Mdn* = 0), T = 26, *p* > 0.05.
- Liked posture continuation (*Mdn* = 2) compared to interesting posture continuation (*Mdn* = 1), T = 86.50, *p* > 0.05.

**Non-significant results (experiment 2)**

**3.2.1 Making Liked and Disliked Sequences**

The Wilcoxon Signed Rank Test showed there were no significant differences between the following comparisons:

- Global symmetry with abstract images for liking judgements (*Mdn* = 0.00) and disliking judgements (*Mdn* = 0.00), *z* = -1.85, *p* > 0.05.

- Continuation produced with abstract images for dislike judgements (*Mdn* = 2.00) and continuation produced with abstract images for liking judgements (*Mdn* = 3.00), z = -1.08, *p* > 0.05.

- Local symmetry produced with postures for dislike judgements (*Mdn* = 0.00) and local symmetry produced with postures for liking judgements (*Mdn* = 0.00), z = -0.16, *p* > 0.05.

- Continuation with postures for liking (*Mdn* = 3.00) and for dislike (*Mdn* = 2.00), *z* = -1.79, *p* > 0.05.

**3.2.2 Making Abstract and Body Posture Sequences**

The Wilcoxon Signed Rank Test showed no significant differences for the following comparisons of aesthetic features produced with abstract images against aesthetic features produced with body postures images:

- Continuation produced with postures for liking judgements (*Mdn* = 3.00) and continuation produced with abstract images for liking judgements (*Mdn* = 3.00), *z* = -0.39, *p* > 0.05.
- Global symmetry produced with postures for liking judgements (*Mdn* = 0.00) and global symmetry produced with abstract images for liking judgements (*Mdn* = 0.00), z = -0.20, *p* > 0.05.
- Local symmetry produced with postures for liking judgements (*Mdn* = 0.00) and local symmetry produced with abstract images for liking judgements (*Mdn* = 0.50), z = -1.20, *p* > 0.05.
- Continuation produced with postures for dislike judgements (*Mdn* = 2.00) and continuation produced with abstract images for dislike judgements (*Mdn* = 2.00), z = -0.58, *p* > 0.05.
- Global symmetry produced with postures for dislike judgements (*Mdn* = 0.00) and global symmetry produced with abstract images for dislike judgements (*Mdn* = 0.00), z = 0.00, p > 0.05.
- Local symmetry produced with postures for dislike judgements (*Mdn* = 0.00) and local symmetry produced with abstract images for dislike judgements (*Mdn* = 0.00), z = -1.11, *p* > 0.05.
