# Supplementary figures and images for: Aesthetic preference in the production of image sequences
Source: Front Psychol. 2023 Nov 30;14:1165143. doi: 10.3389/fpsyg.2023.1165143 (PMC10720618; doi:10.3389/fpsyg.2023.1165143)

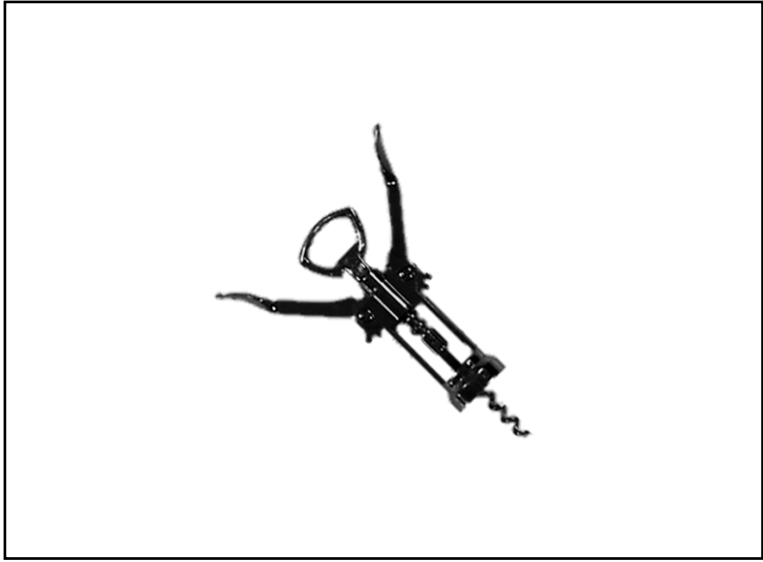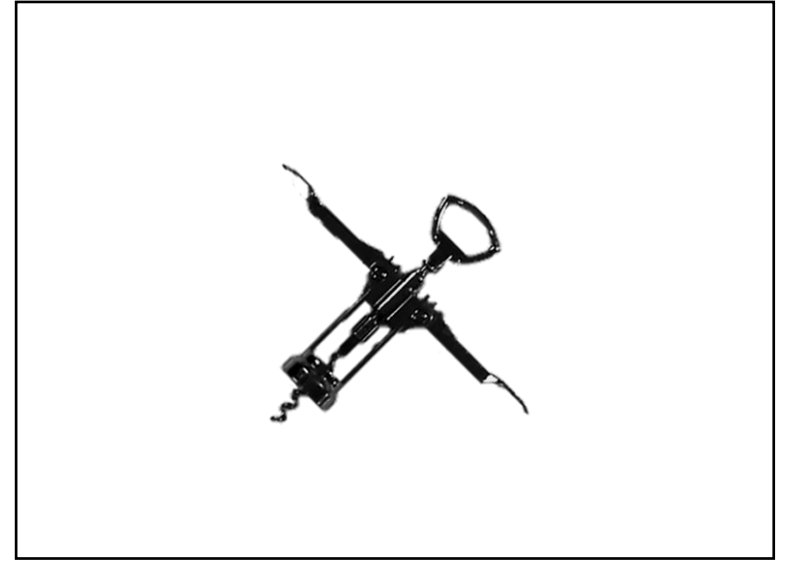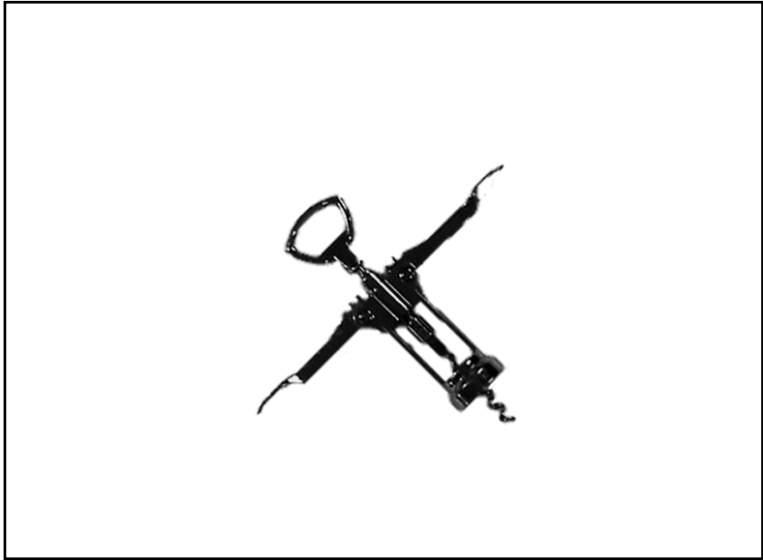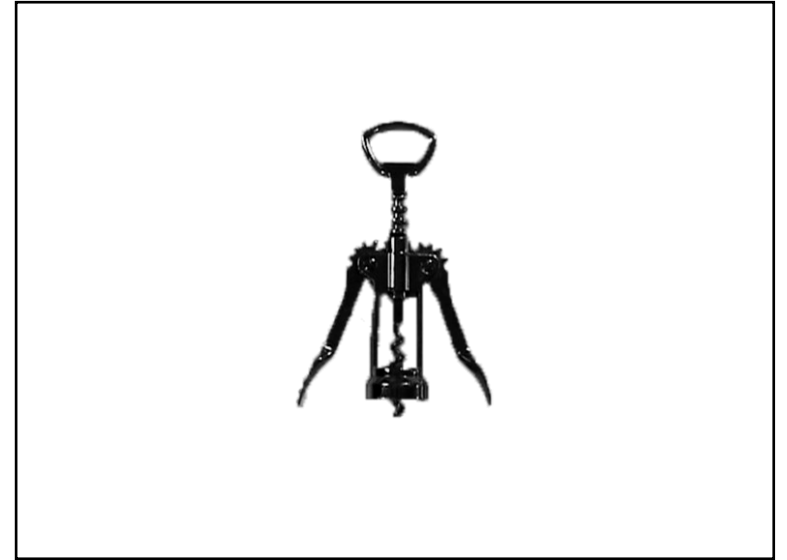

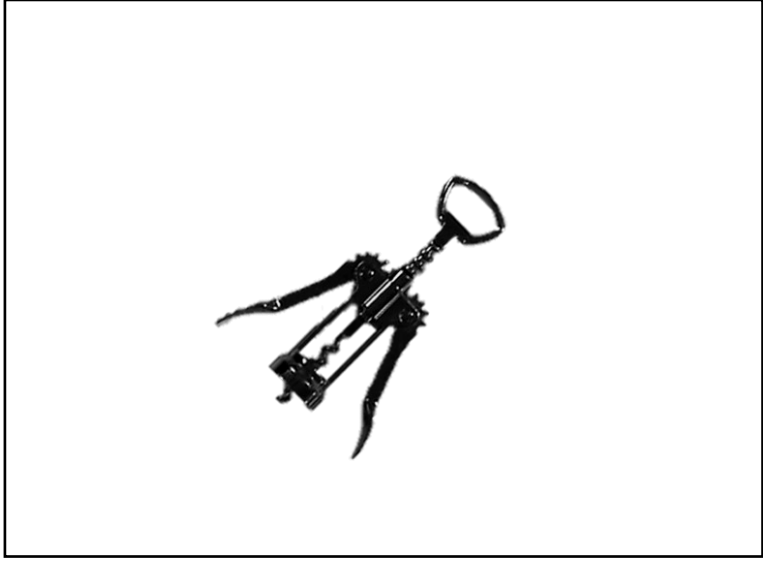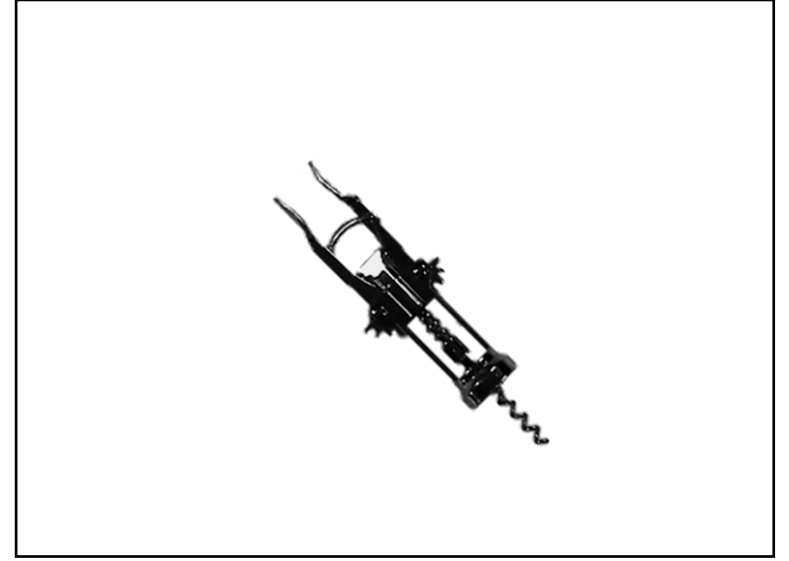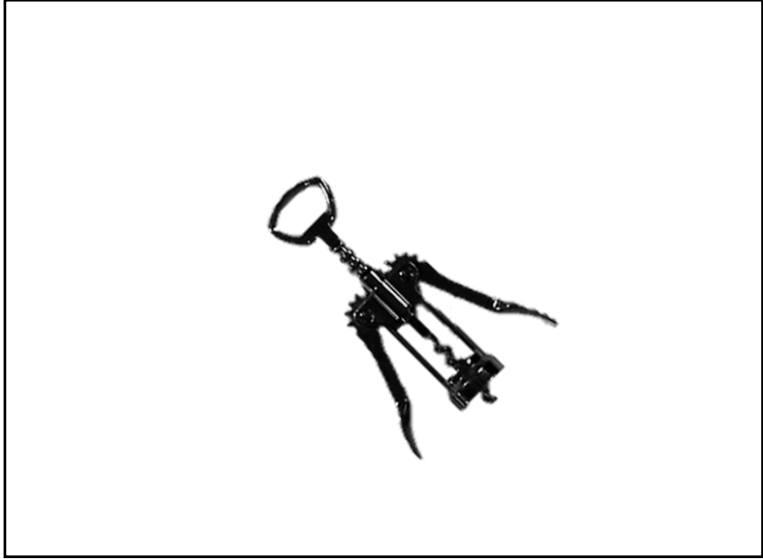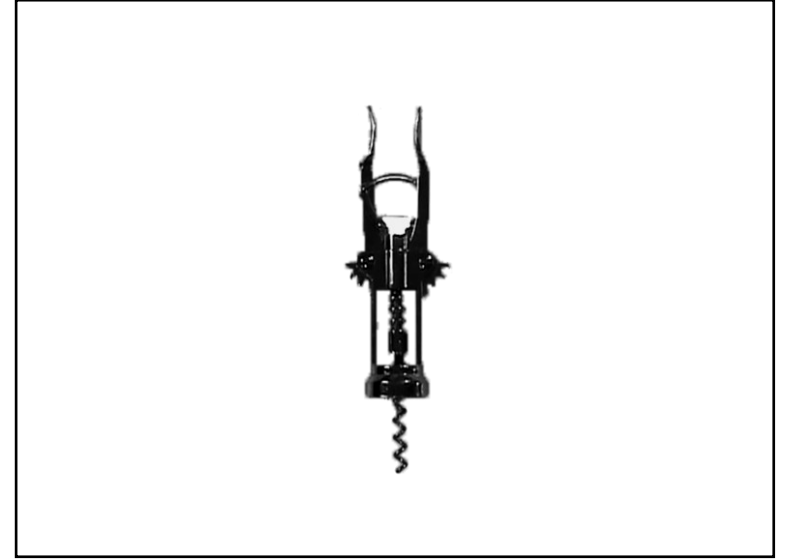

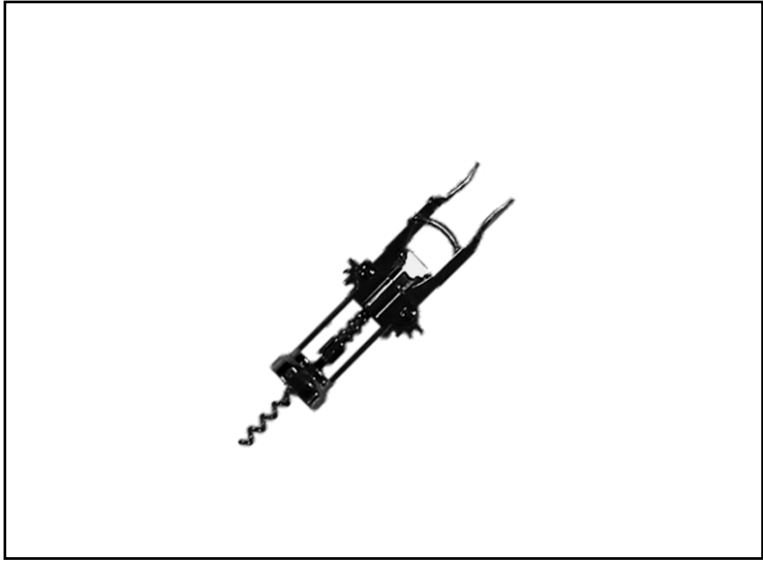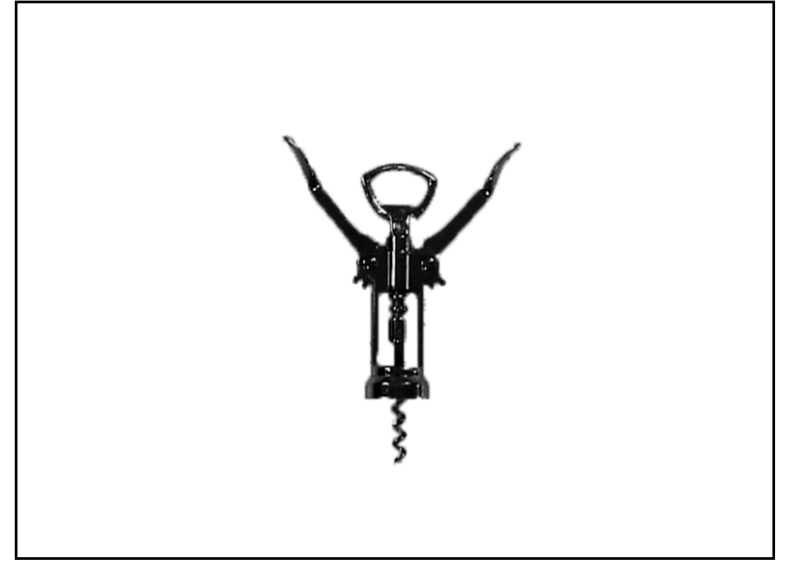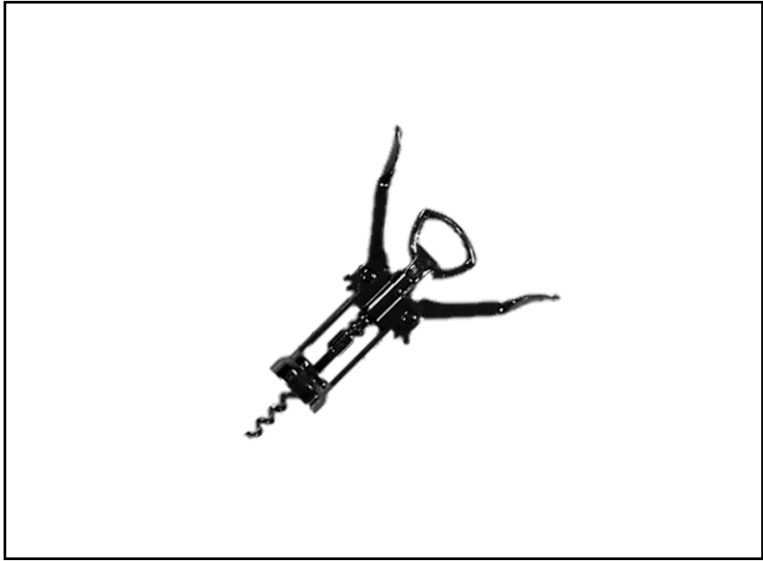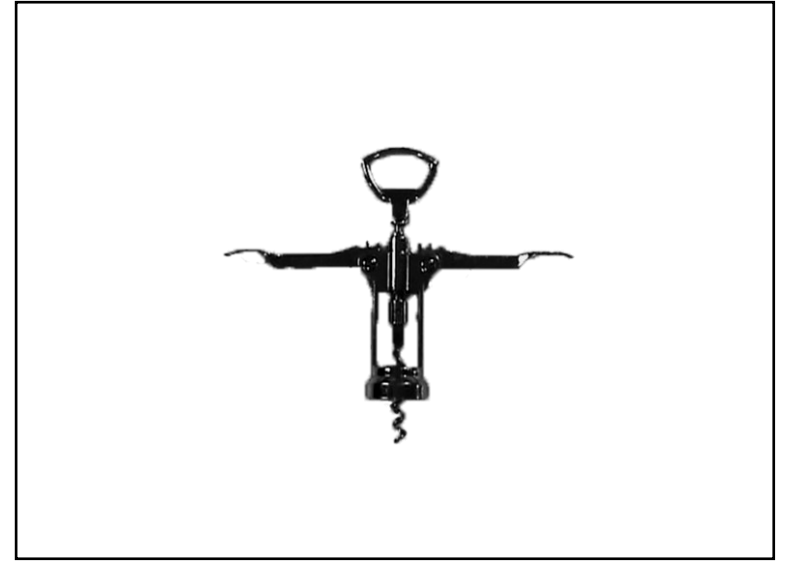

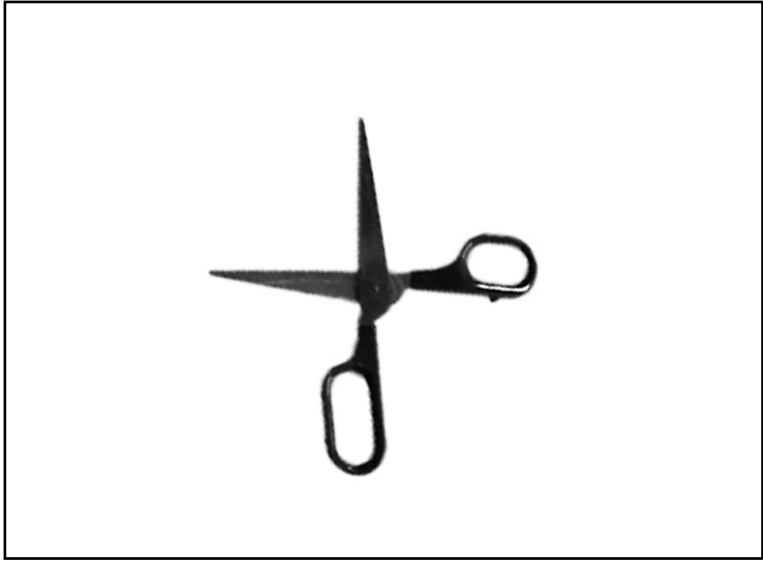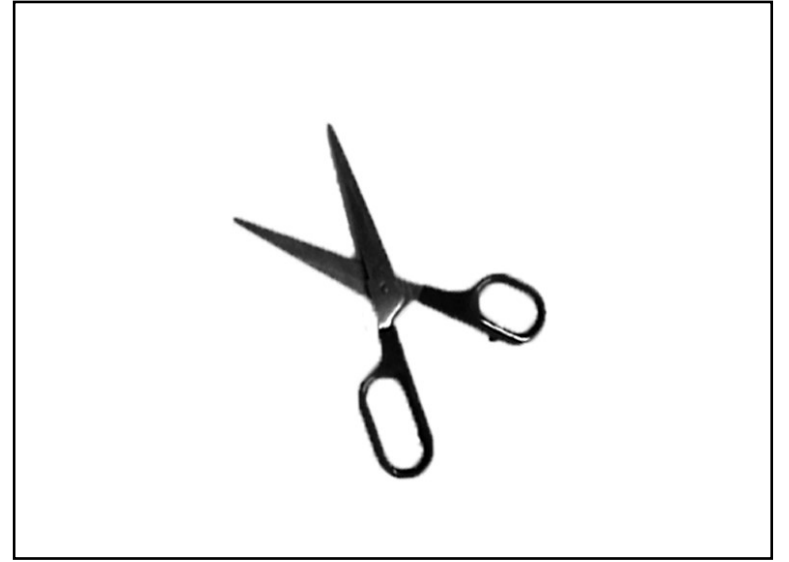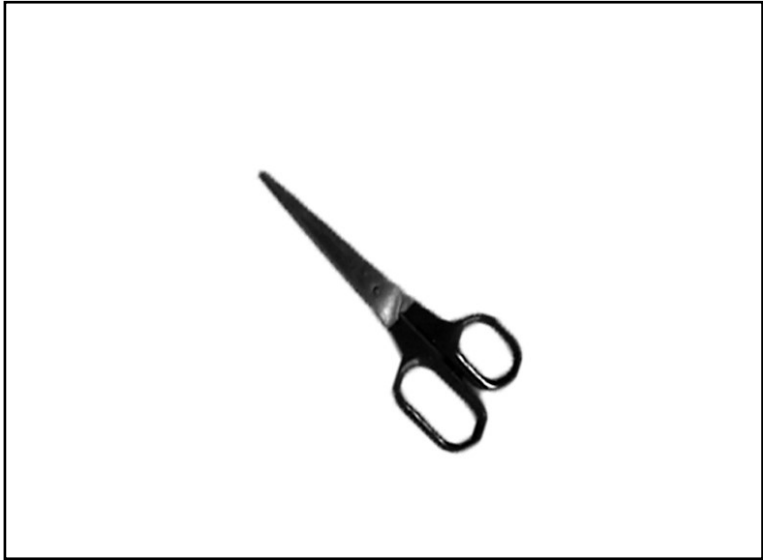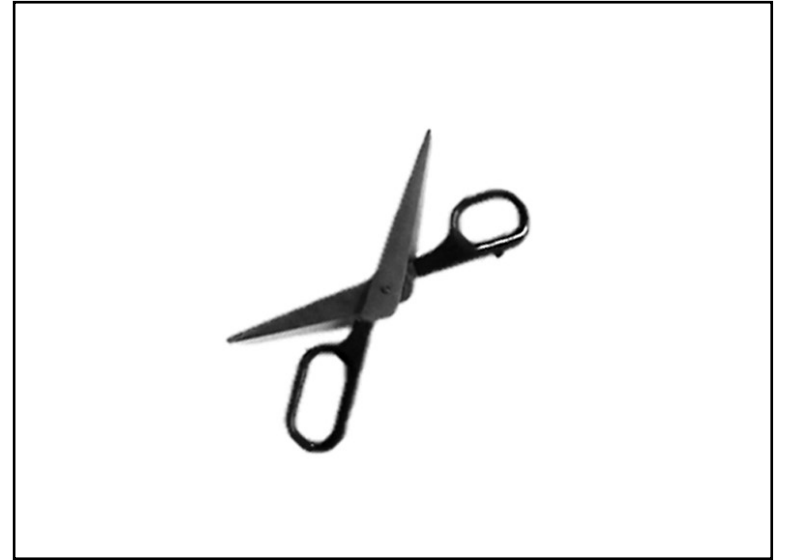

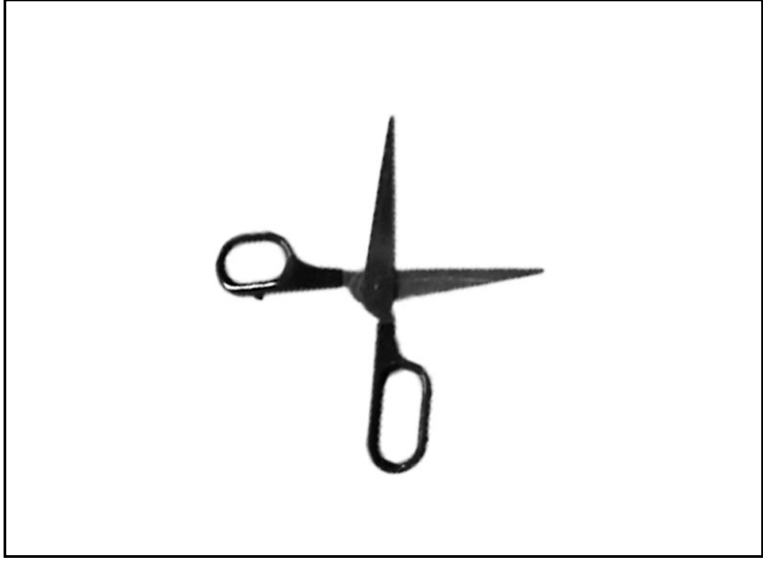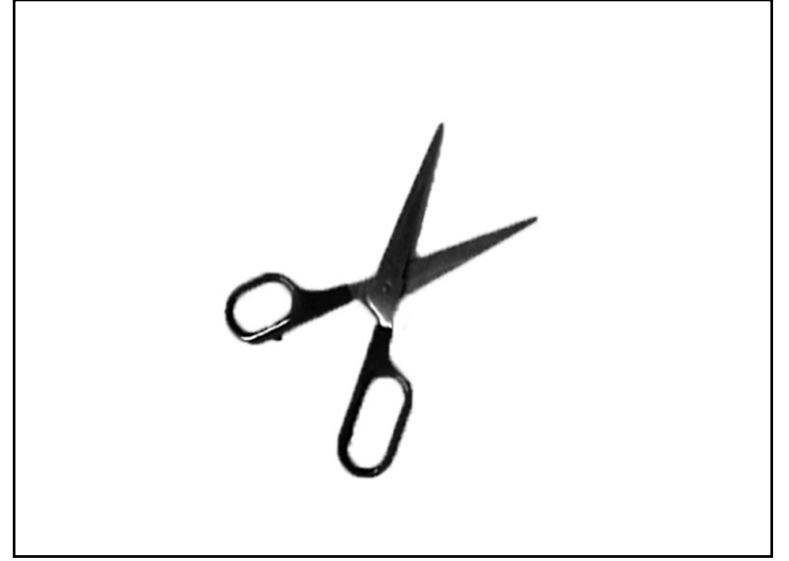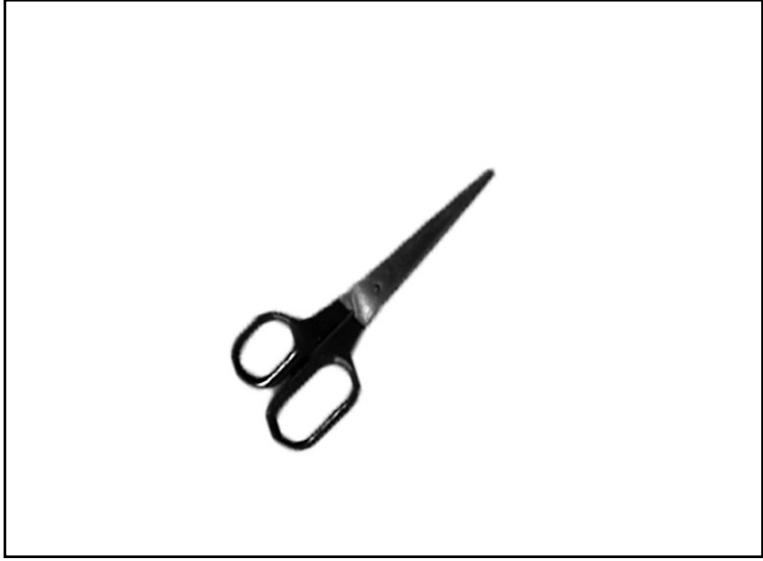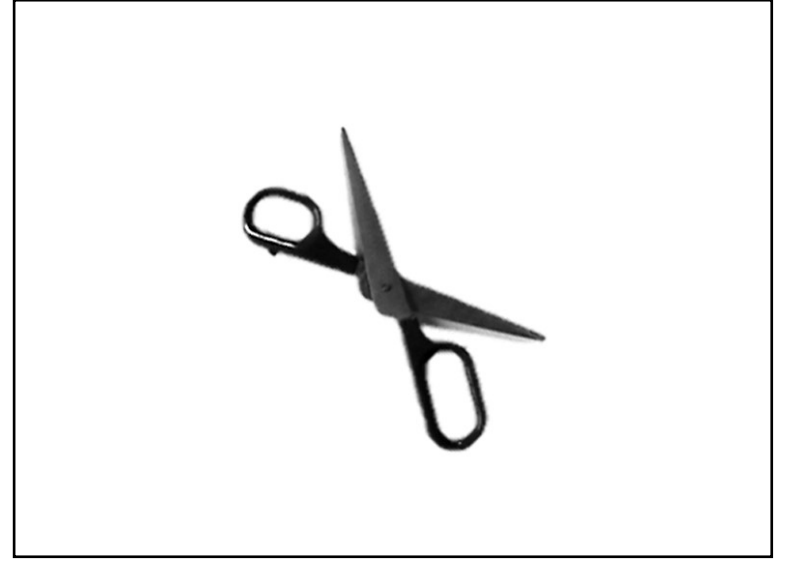

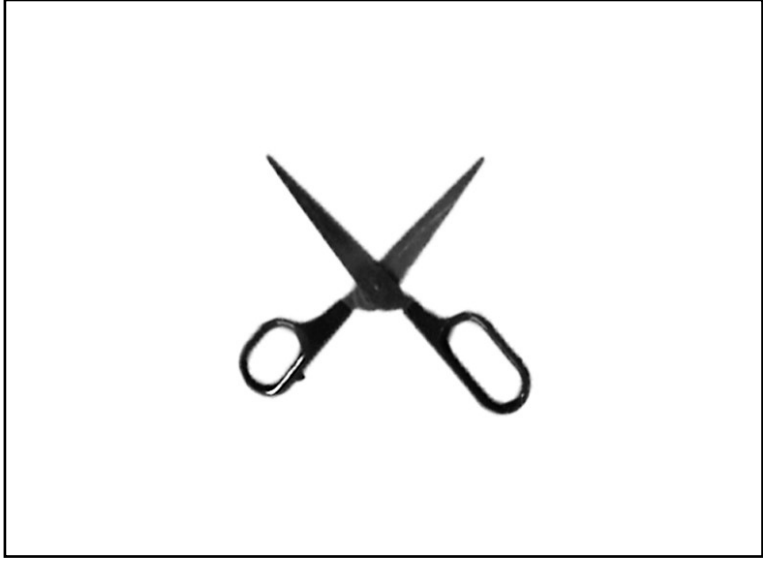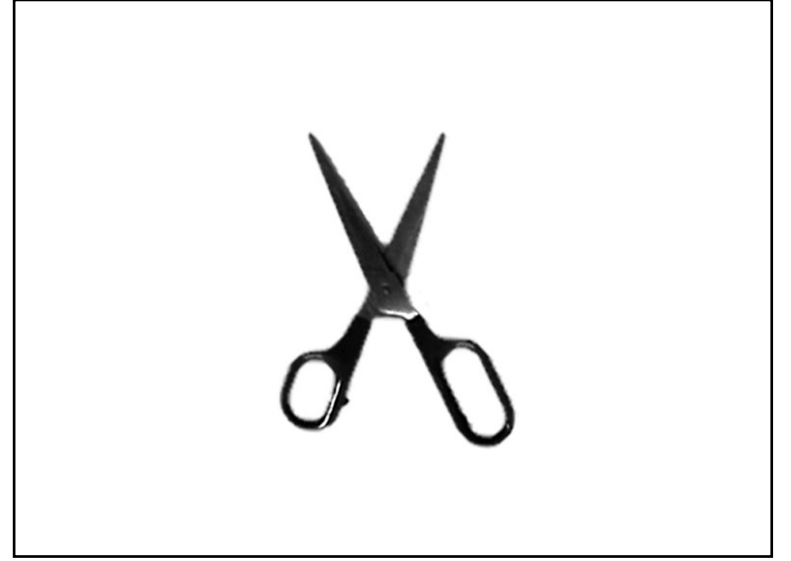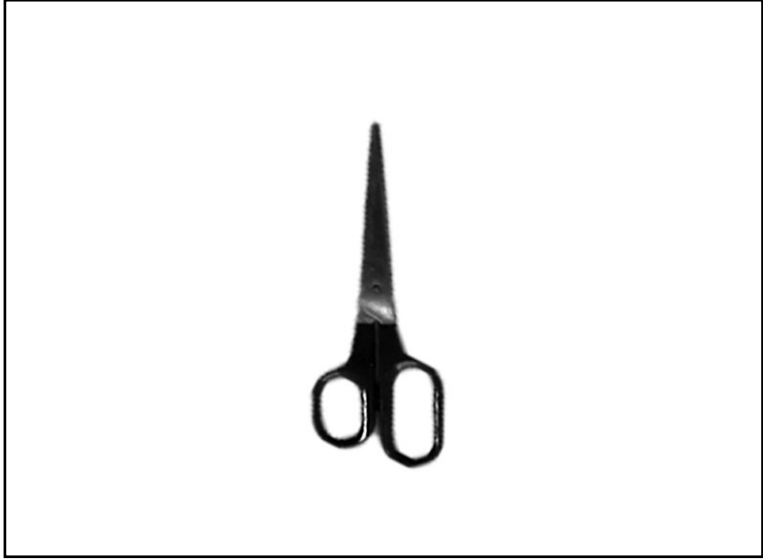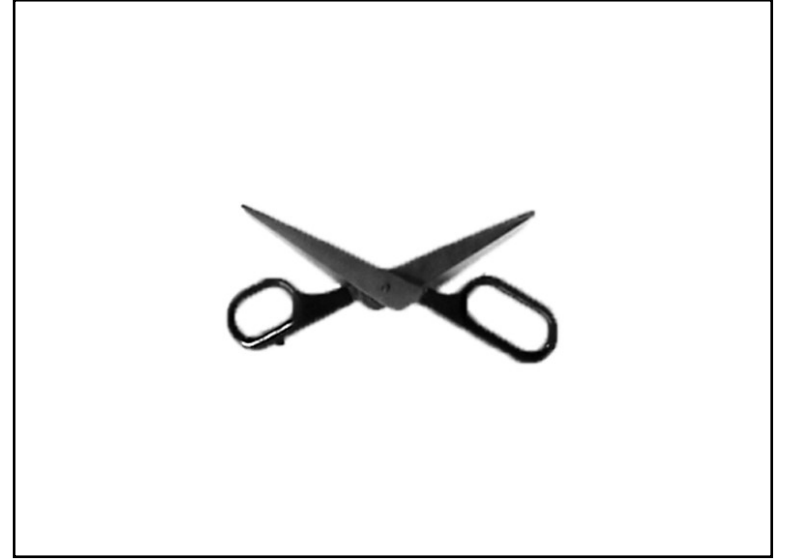

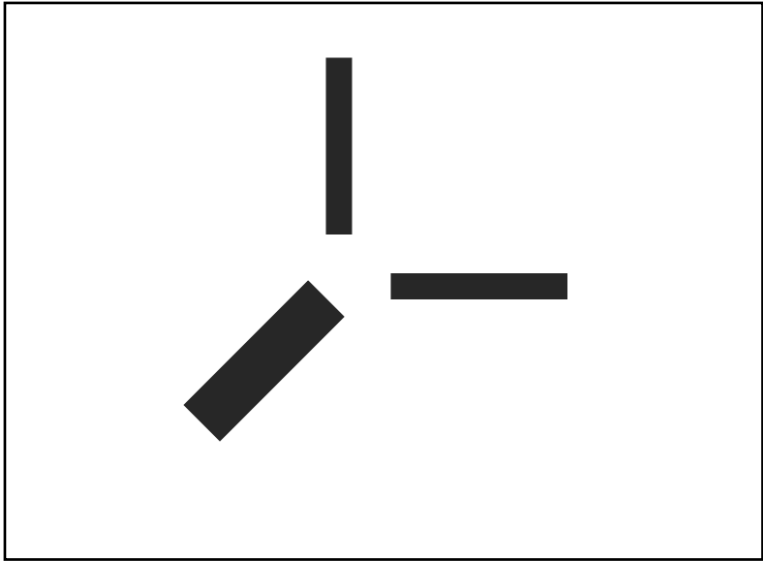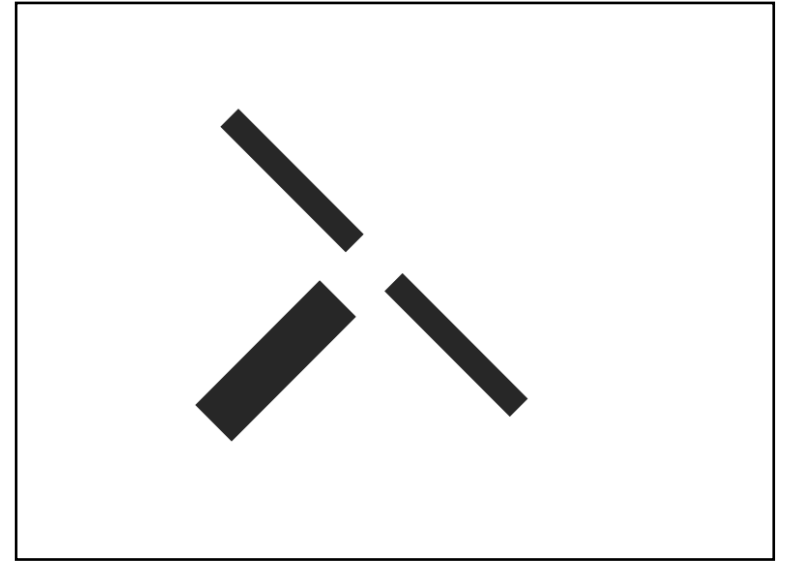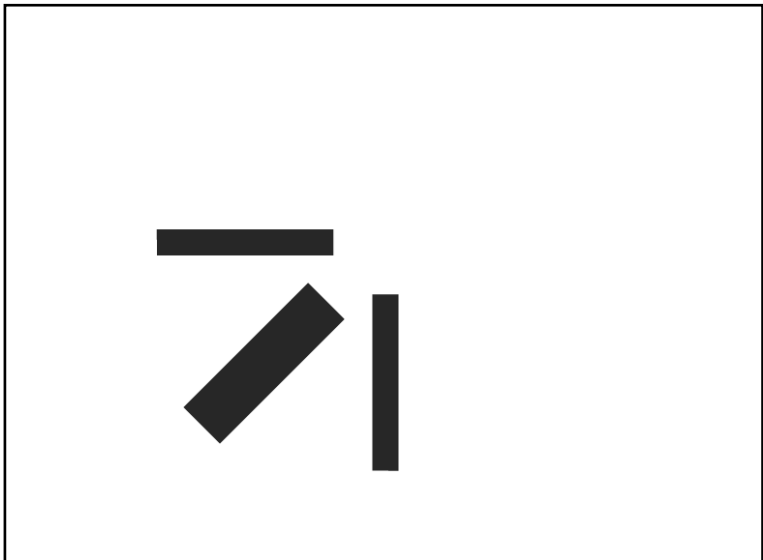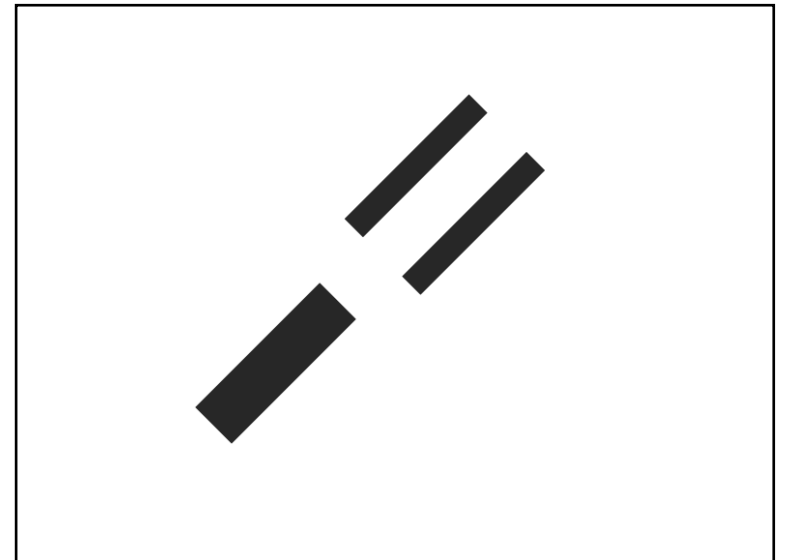

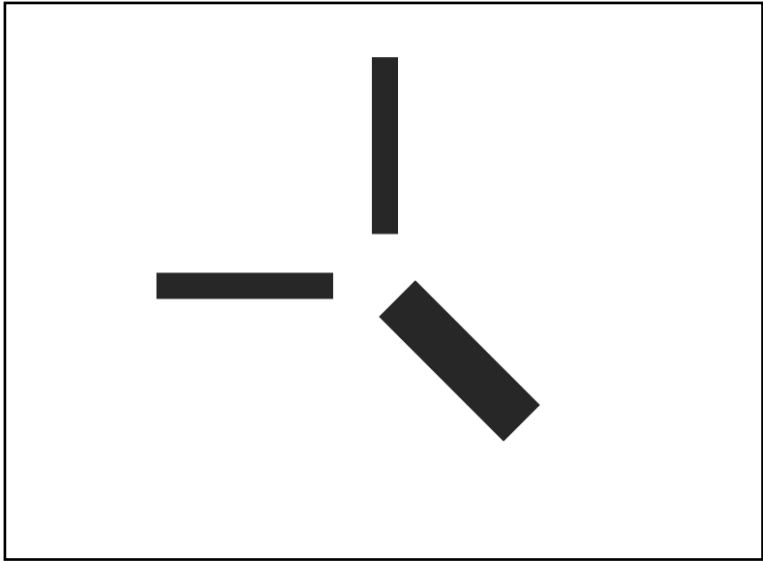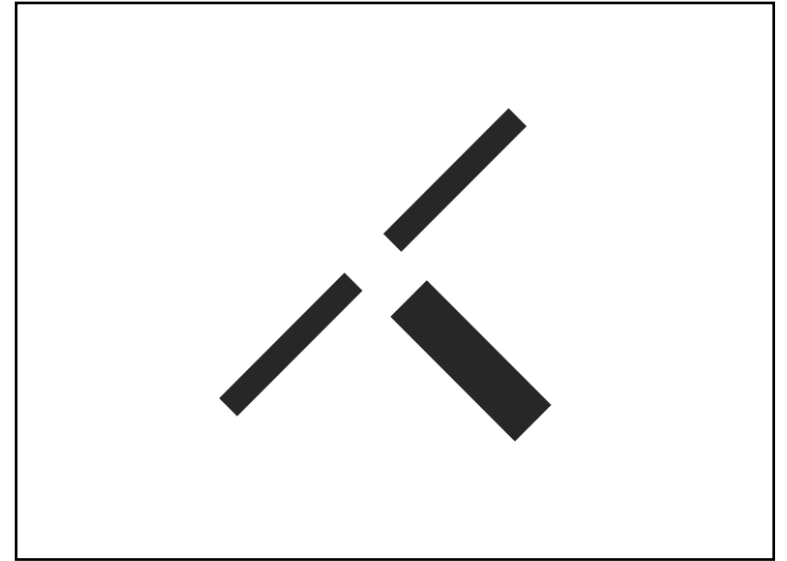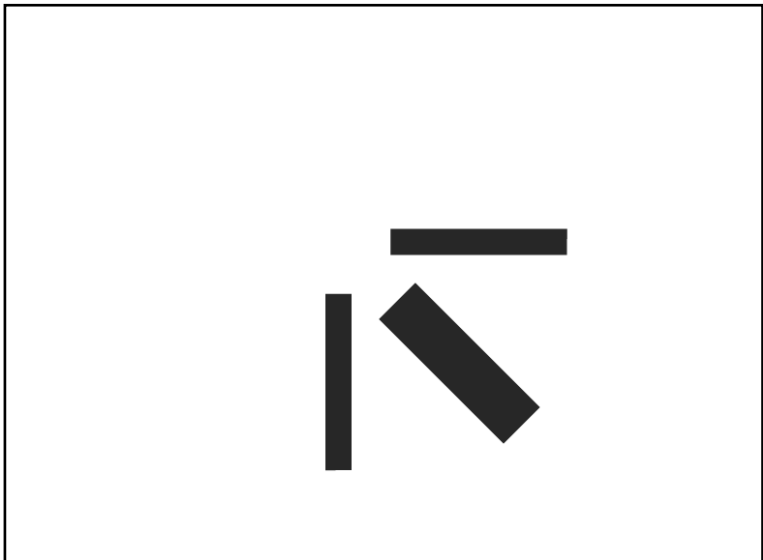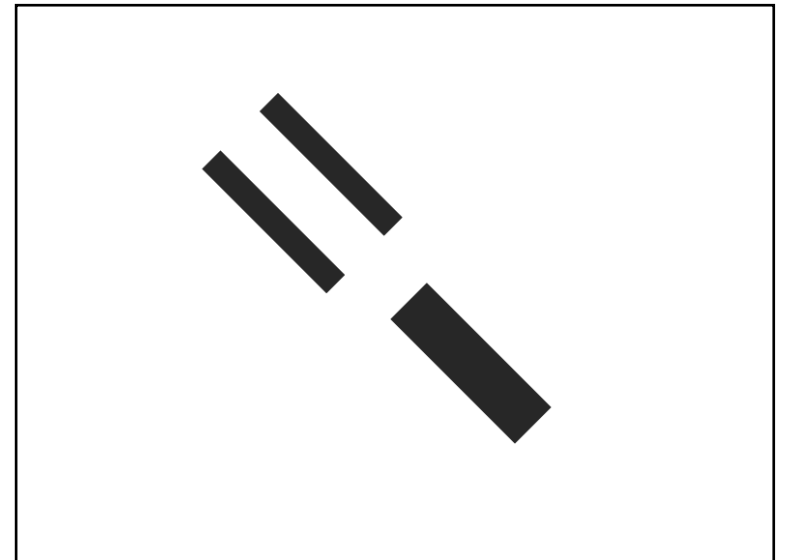

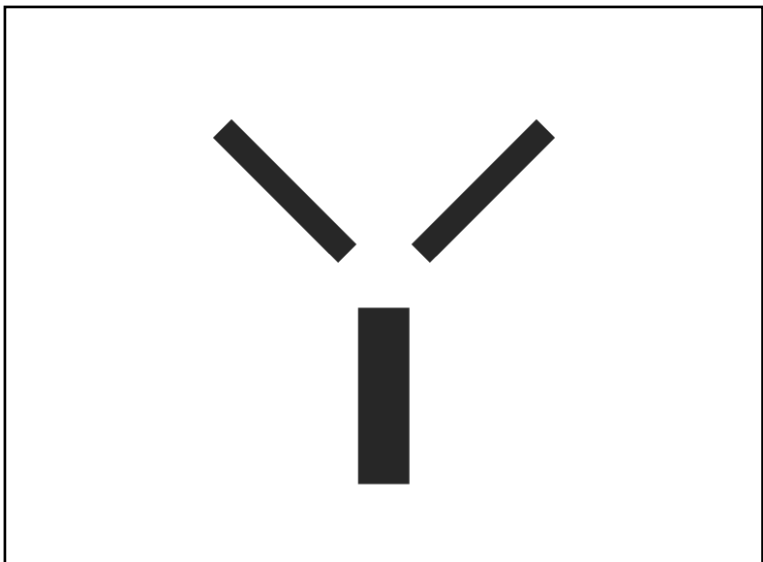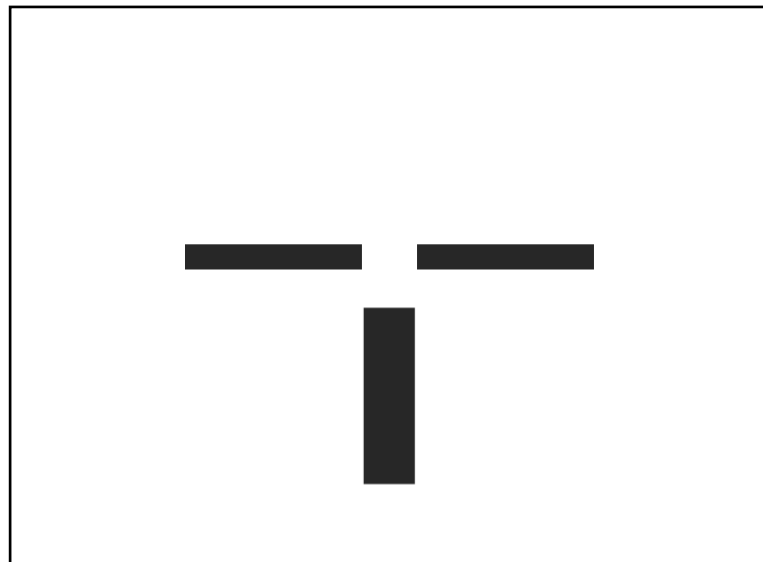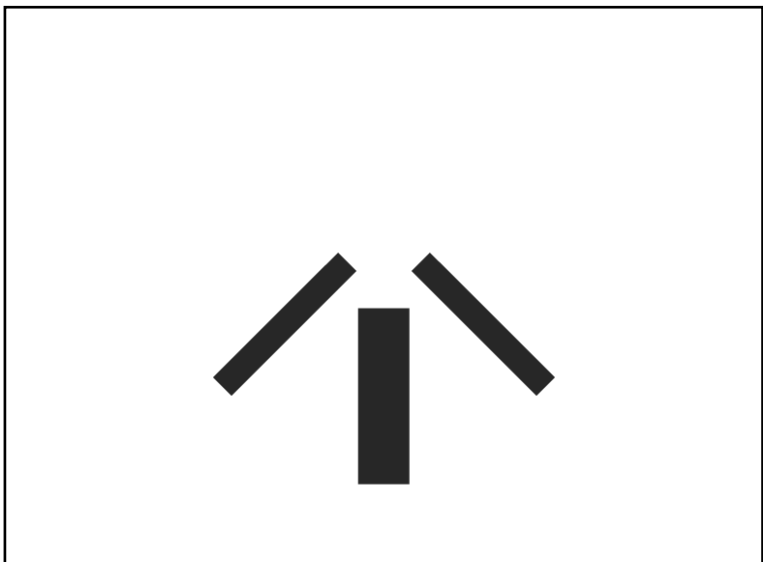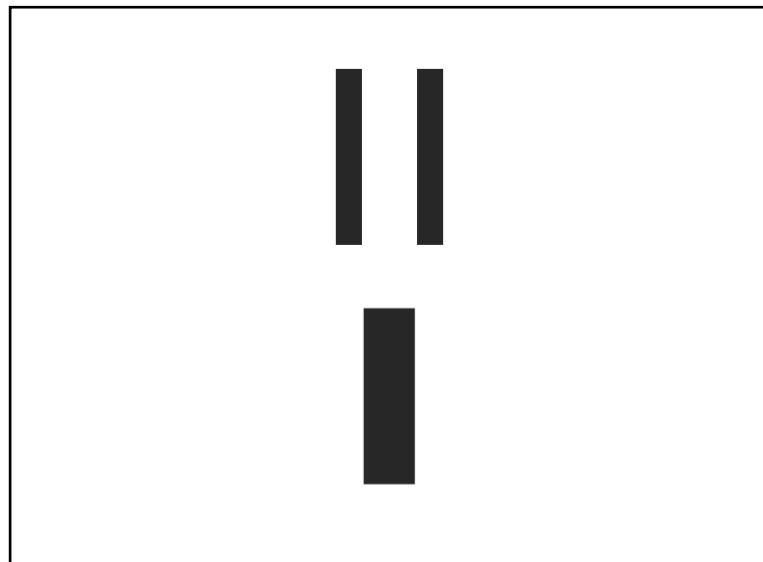

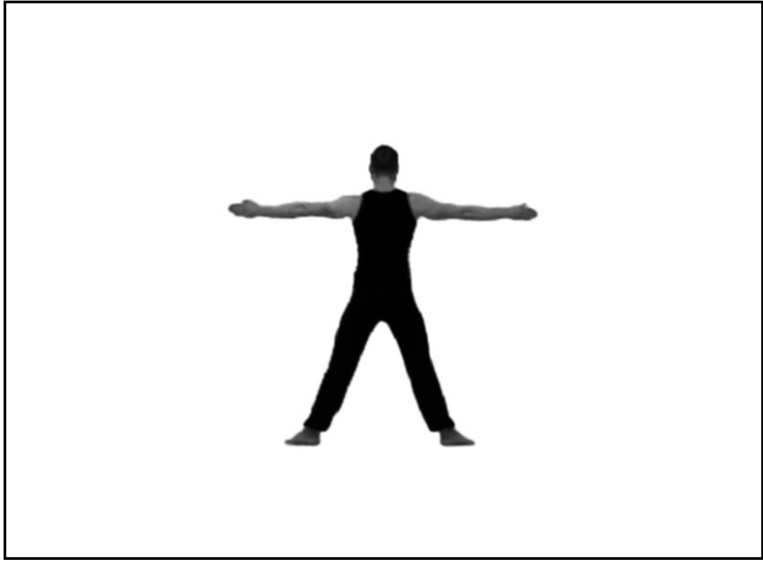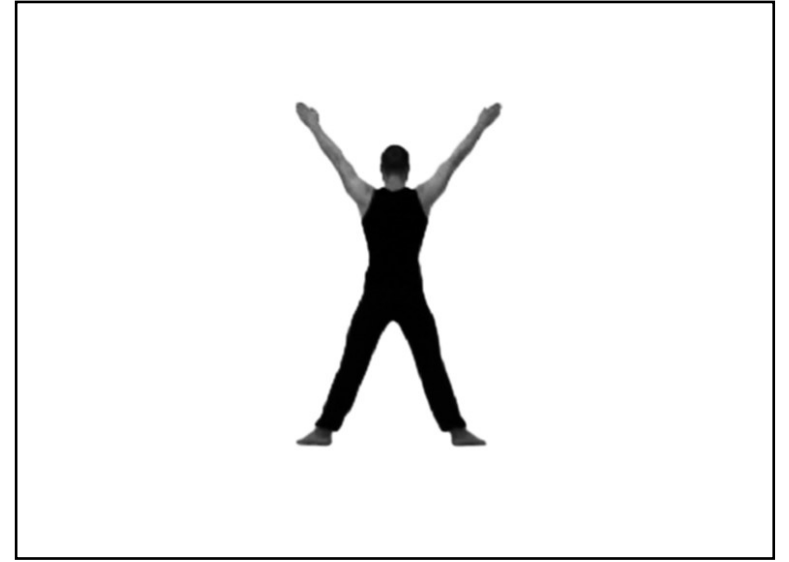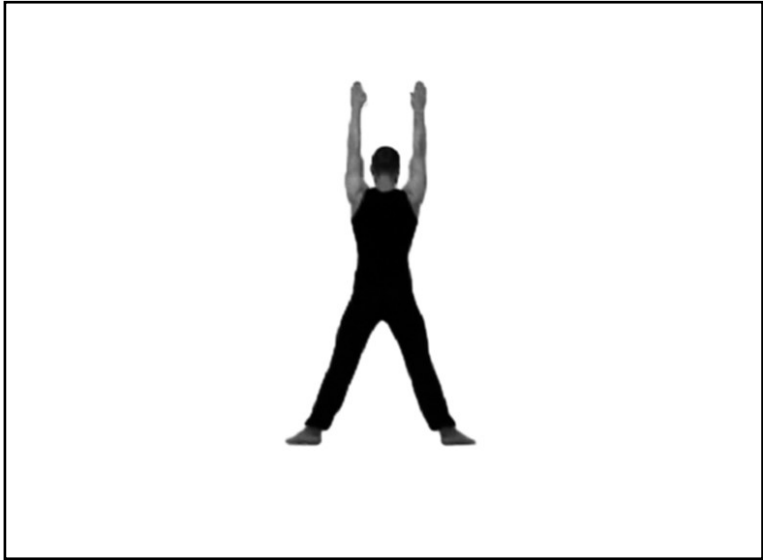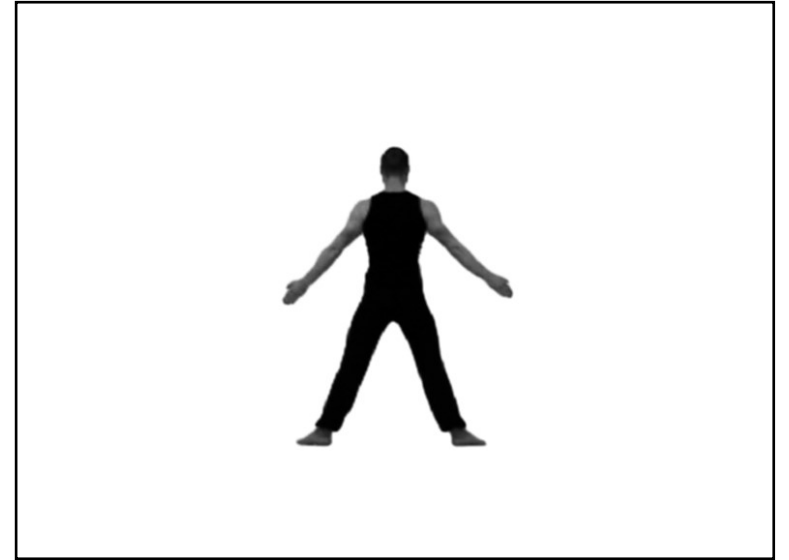

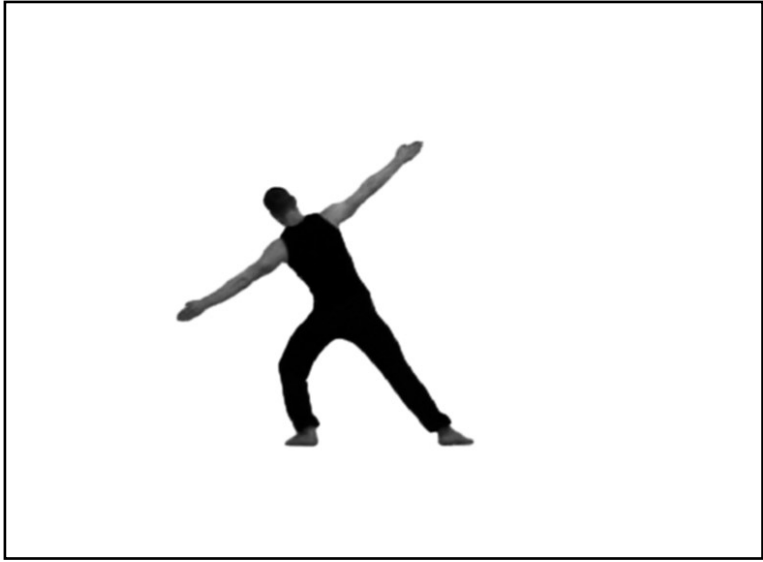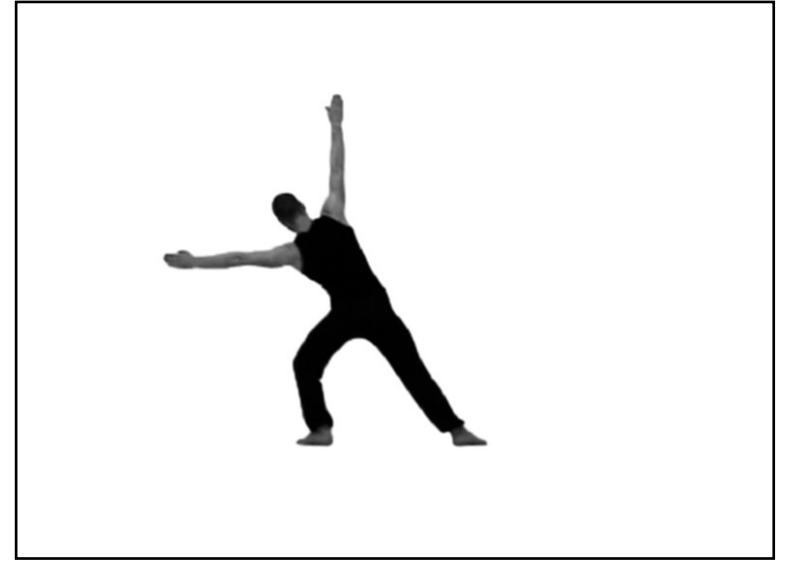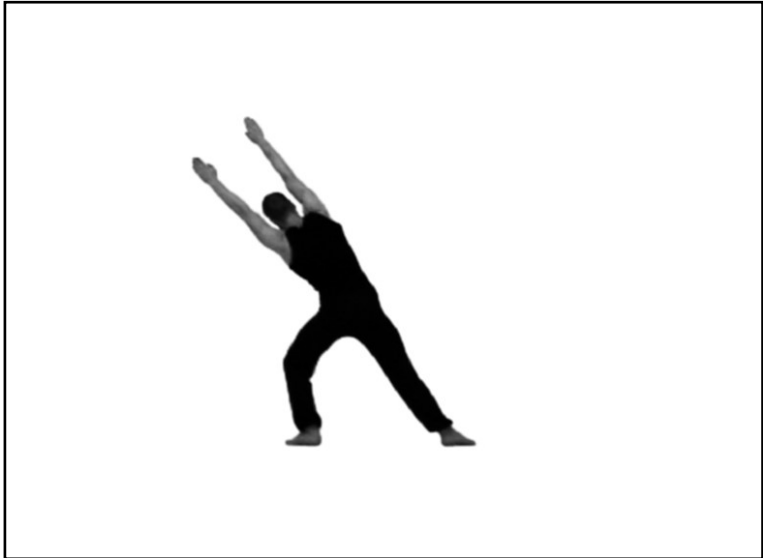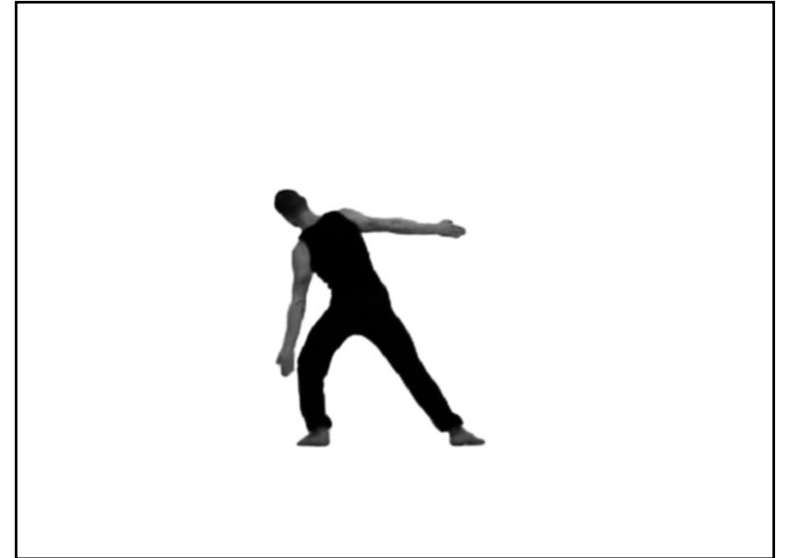

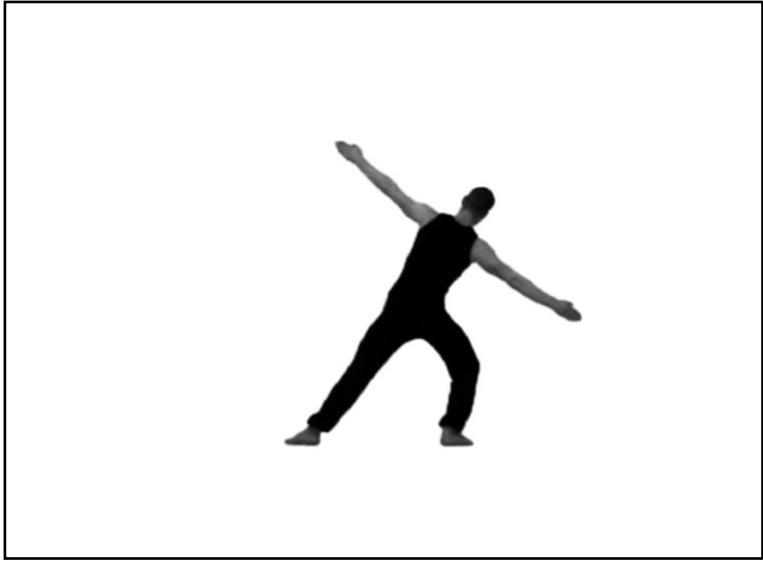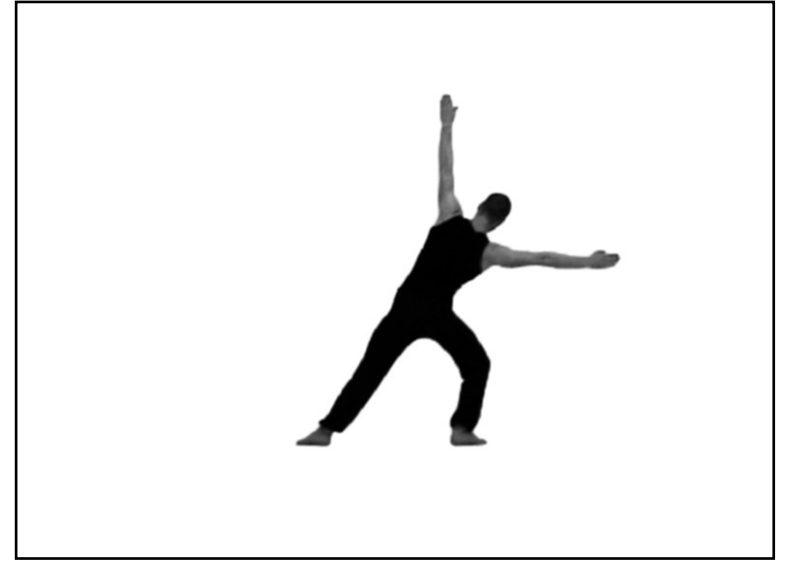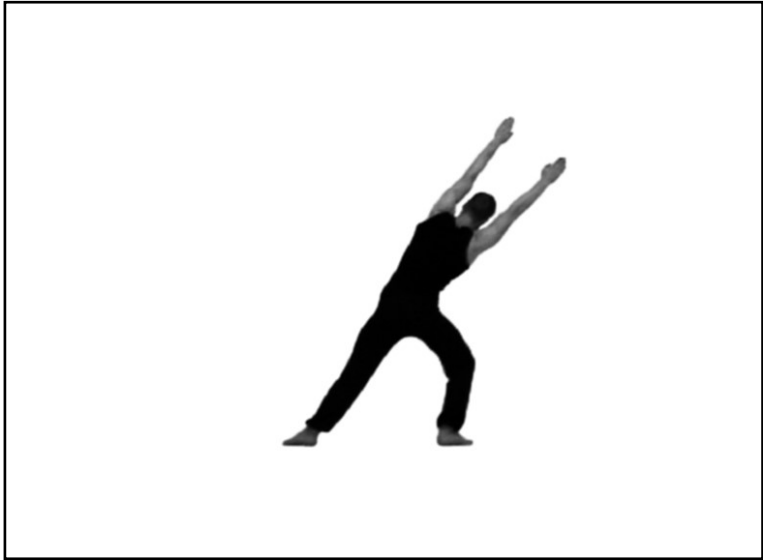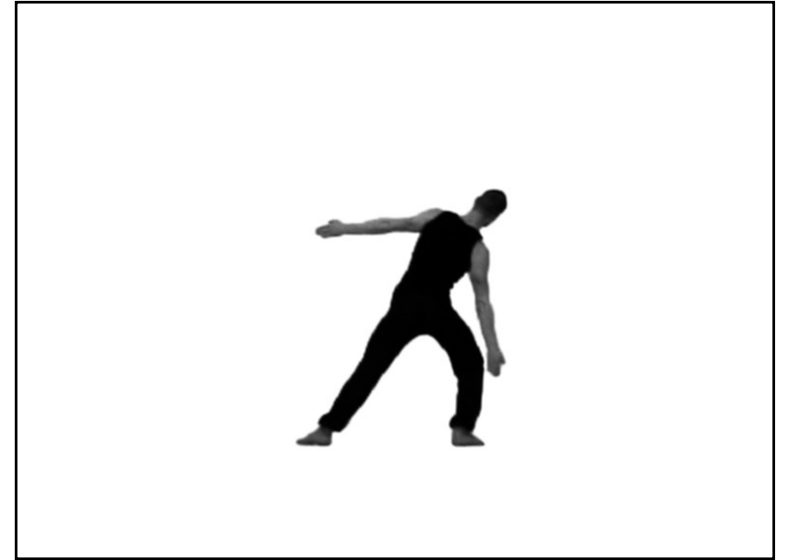

Supplement: Supplementary file 2 [file Data_Sheet_1.PDF]

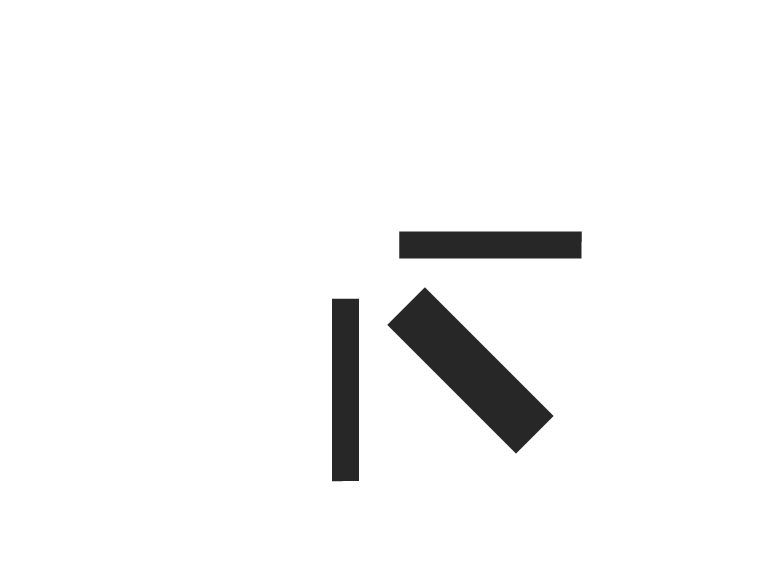

Supplement: Supplementary file 3 [file Data_Sheet_2.ZIP › Experiment 2 images/Abstract/left 1.png]

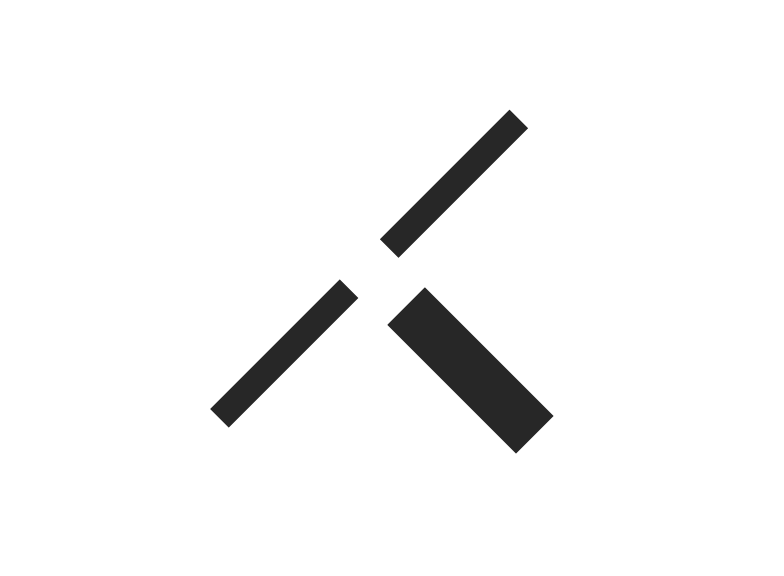

Supplement: Supplementary file 3 [file Data_Sheet_2.ZIP › Experiment 2 images/Abstract/left 2.png]

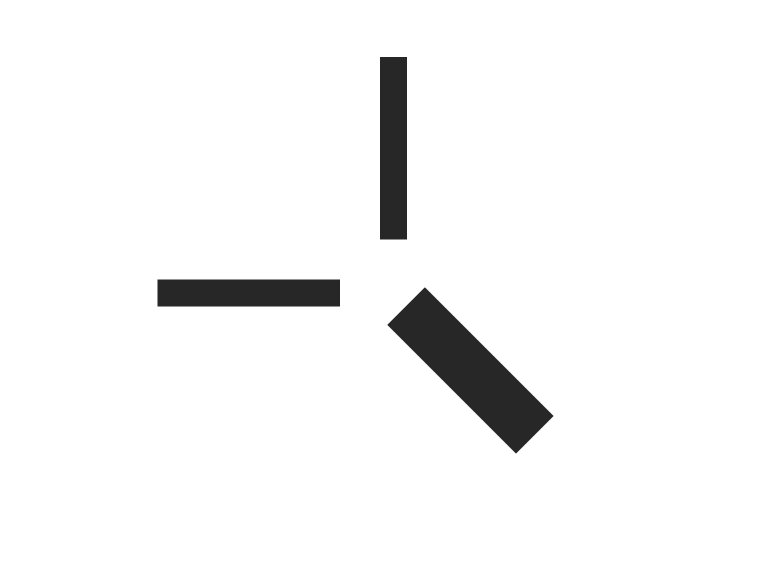

Supplement: Supplementary file 3 [file Data_Sheet_2.ZIP › Experiment 2 images/Abstract/left 3.png]

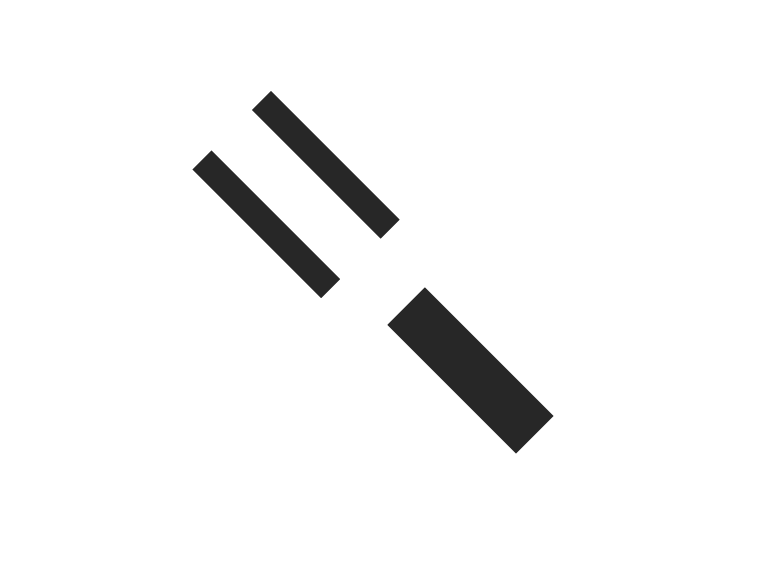

Supplement: Supplementary file 3 [file Data_Sheet_2.ZIP › Experiment 2 images/Abstract/left 4.png]

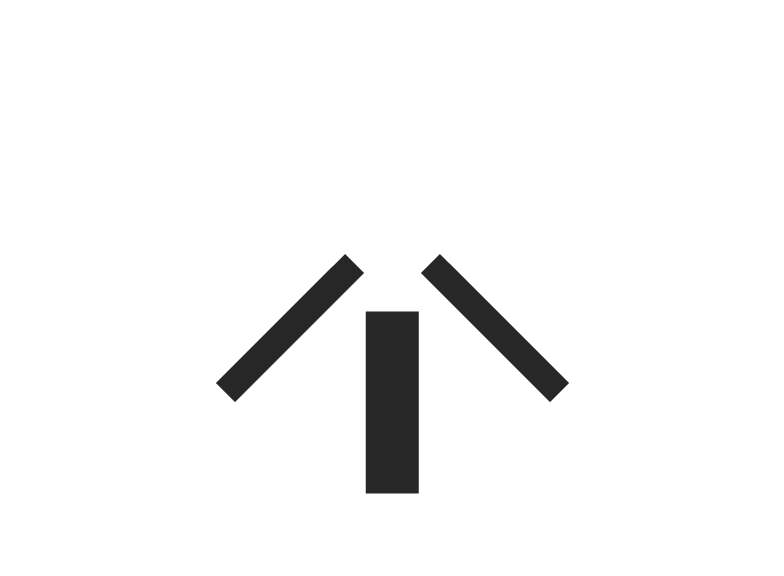

Supplement: Supplementary file 3 [file Data_Sheet_2.ZIP › Experiment 2 images/Abstract/middle 1.png]

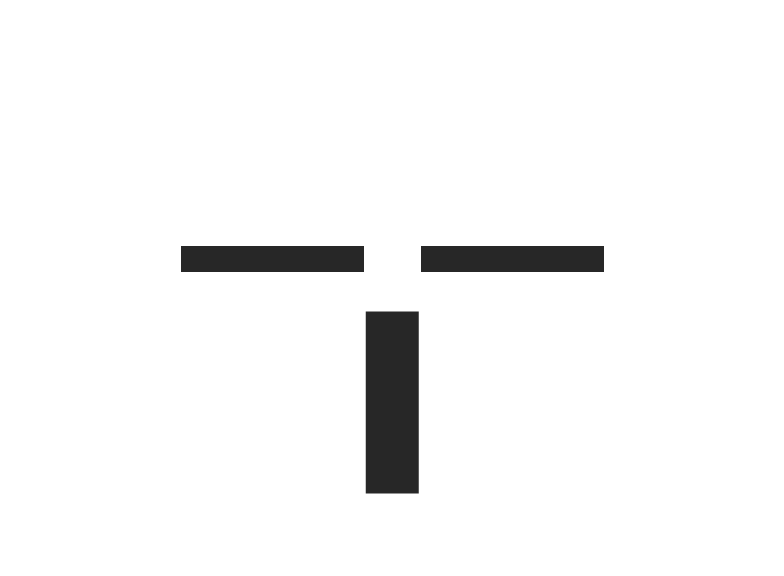

Supplement: Supplementary file 3 [file Data_Sheet_2.ZIP › Experiment 2 images/Abstract/middle 2.png]

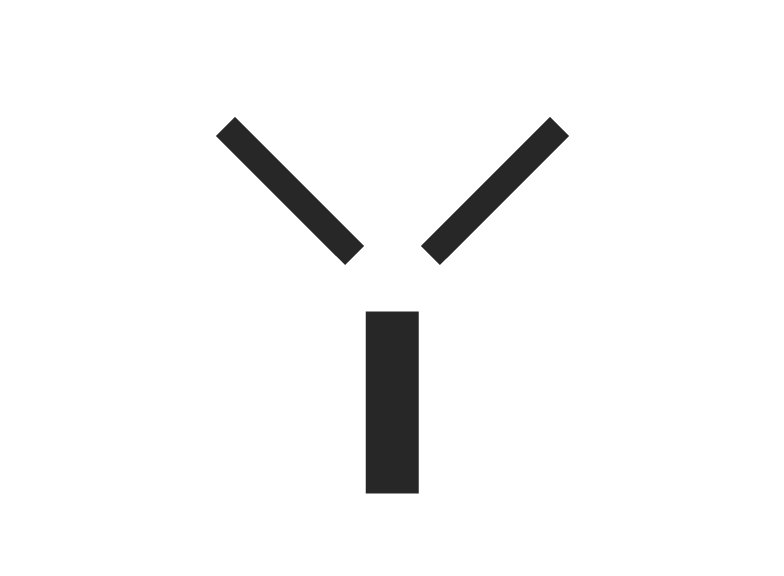

Supplement: Supplementary file 3 [file Data_Sheet_2.ZIP › Experiment 2 images/Abstract/middle 3.png]

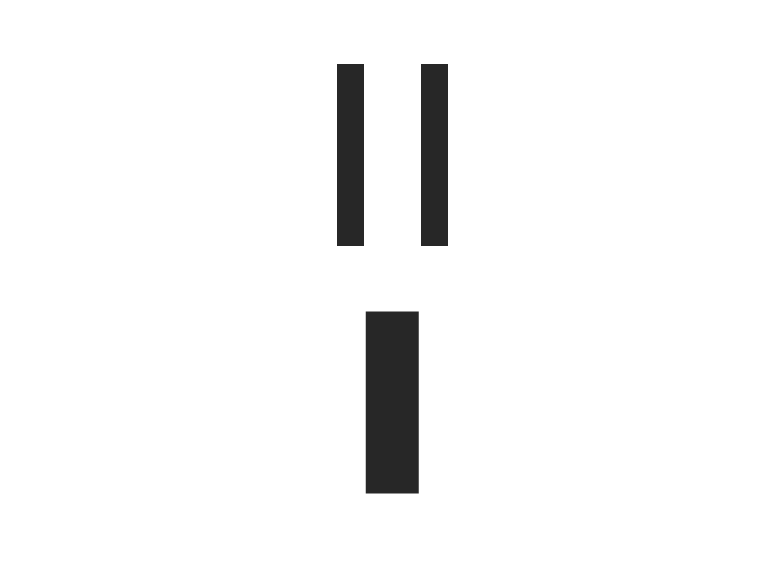

Supplement: Supplementary file 3 [file Data_Sheet_2.ZIP › Experiment 2 images/Abstract/middle 4.png]

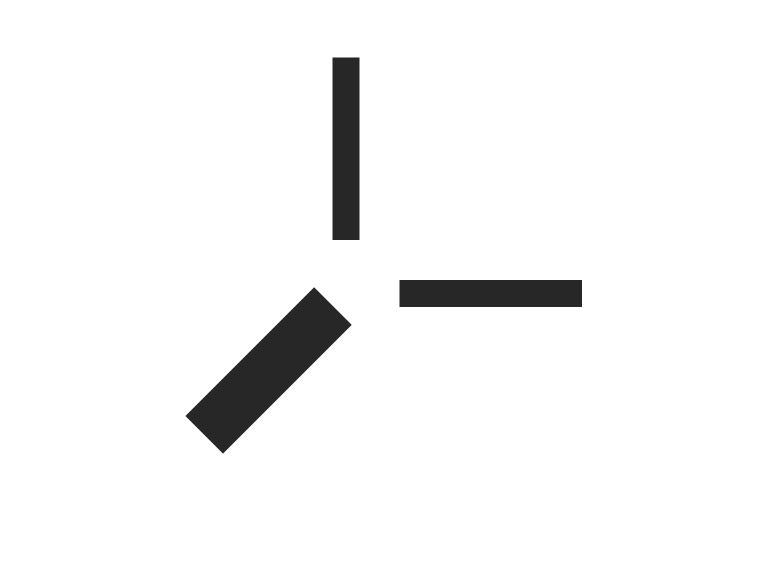

Supplement: Supplementary file 3 [file Data_Sheet_2.ZIP › Experiment 2 images/Abstract/right 03.png]

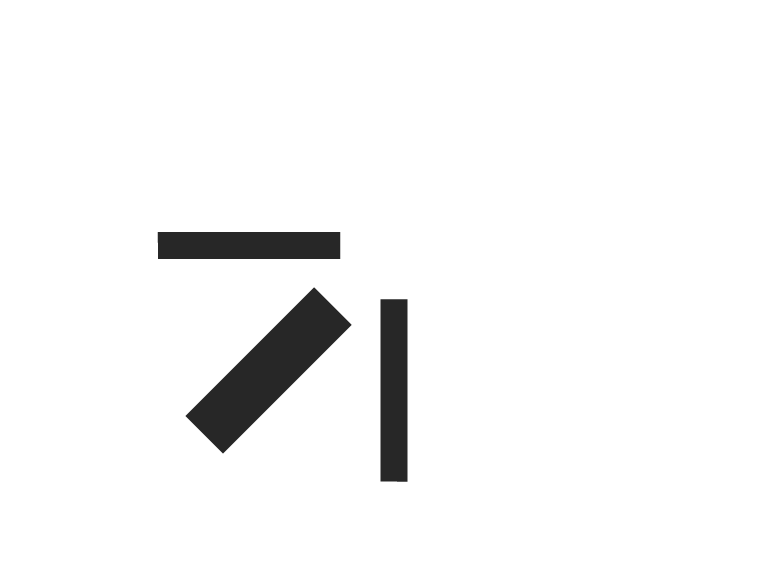

Supplement: Supplementary file 3 [file Data_Sheet_2.ZIP › Experiment 2 images/Abstract/right 1.png]

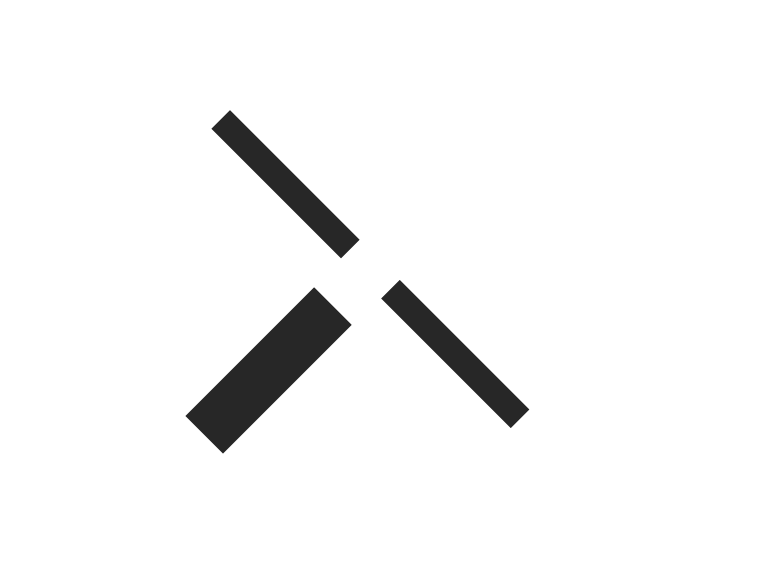

Supplement: Supplementary file 3 [file Data_Sheet_2.ZIP › Experiment 2 images/Abstract/right 2.png]

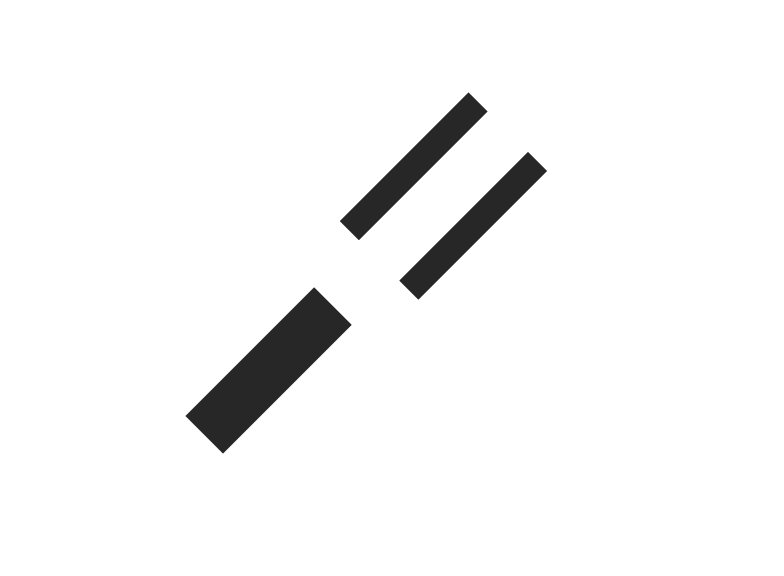

Supplement: Supplementary file 3 [file Data_Sheet_2.ZIP › Experiment 2 images/Abstract/right 4.png]

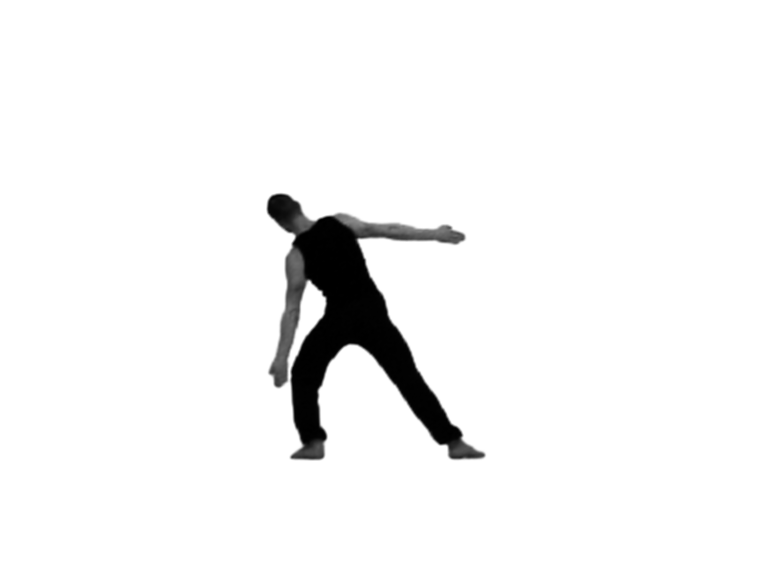

Supplement: Supplementary file 3 [file Data_Sheet_2.ZIP › Experiment 2 images/Postures/left 1.png]

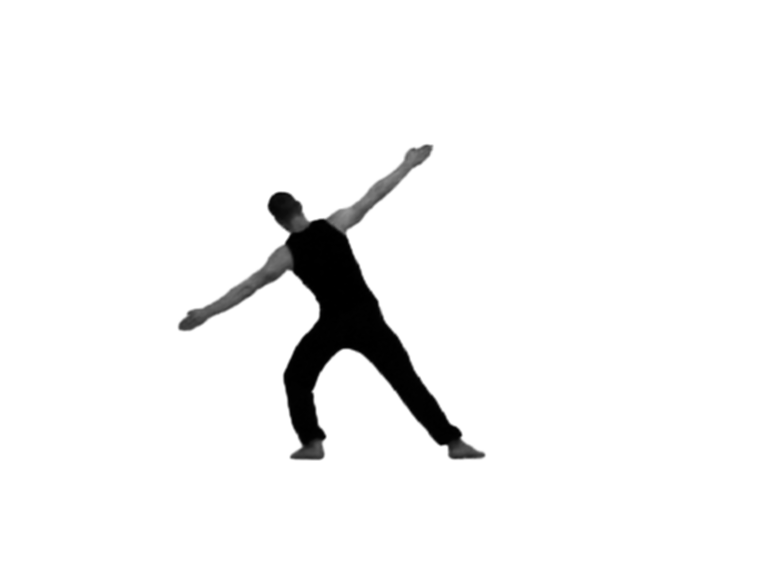

Supplement: Supplementary file 3 [file Data_Sheet_2.ZIP › Experiment 2 images/Postures/left 2.png]

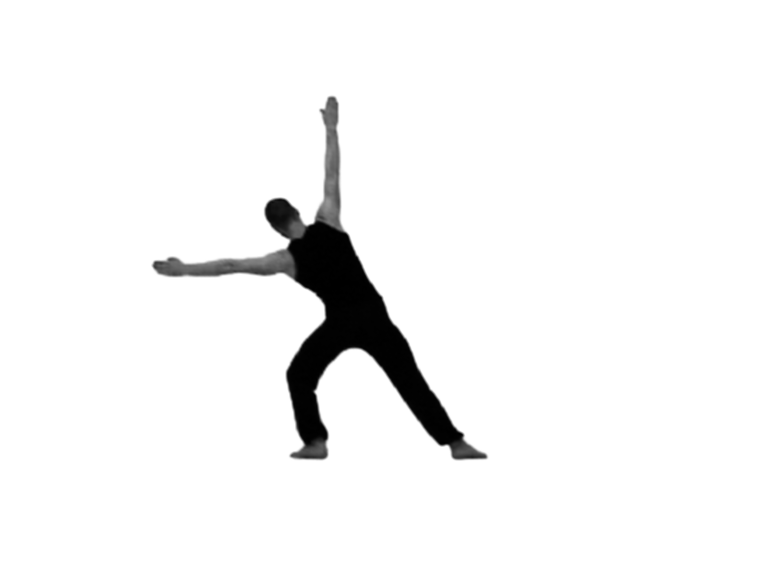

Supplement: Supplementary file 3 [file Data_Sheet_2.ZIP › Experiment 2 images/Postures/left 3.png]

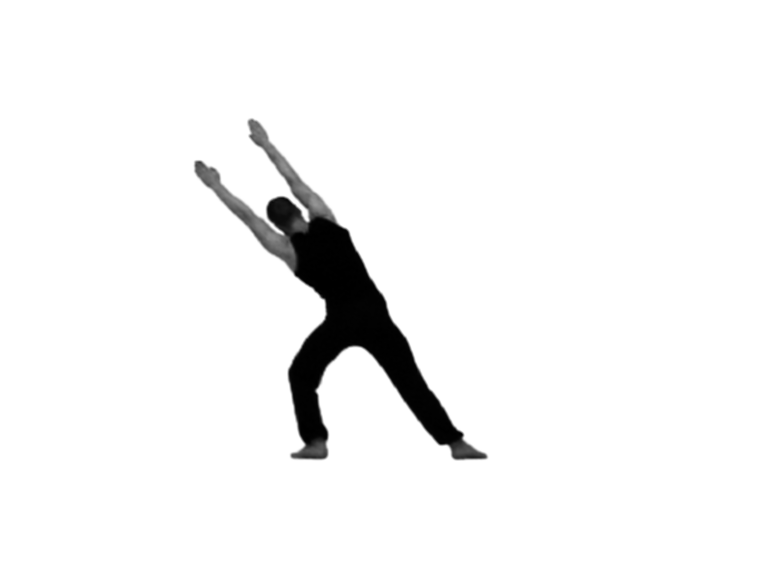

Supplement: Supplementary file 3 [file Data_Sheet_2.ZIP › Experiment 2 images/Postures/left 4.png]

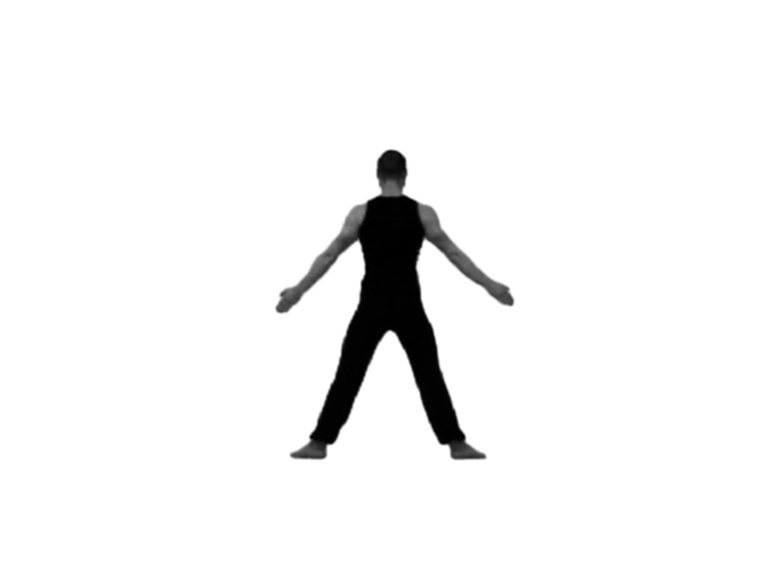

Supplement: Supplementary file 3 [file Data_Sheet_2.ZIP › Experiment 2 images/Postures/middle 1.png]

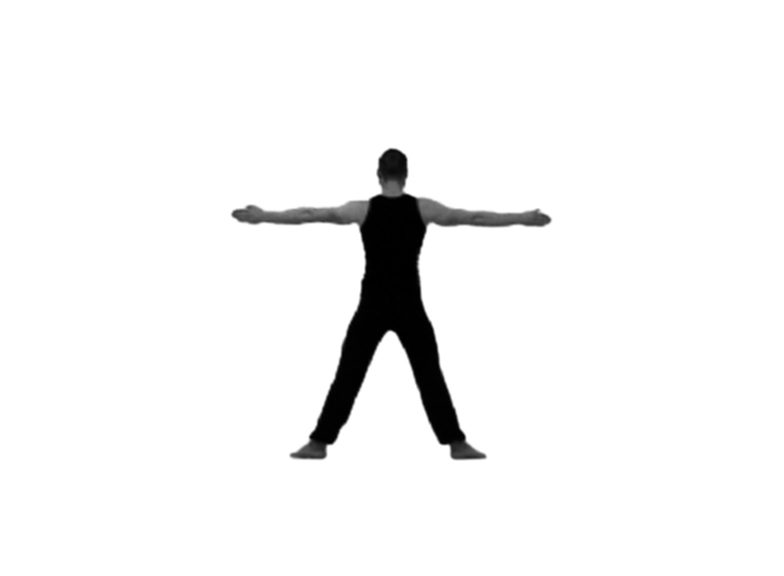

Supplement: Supplementary file 3 [file Data_Sheet_2.ZIP › Experiment 2 images/Postures/middle 2.png]

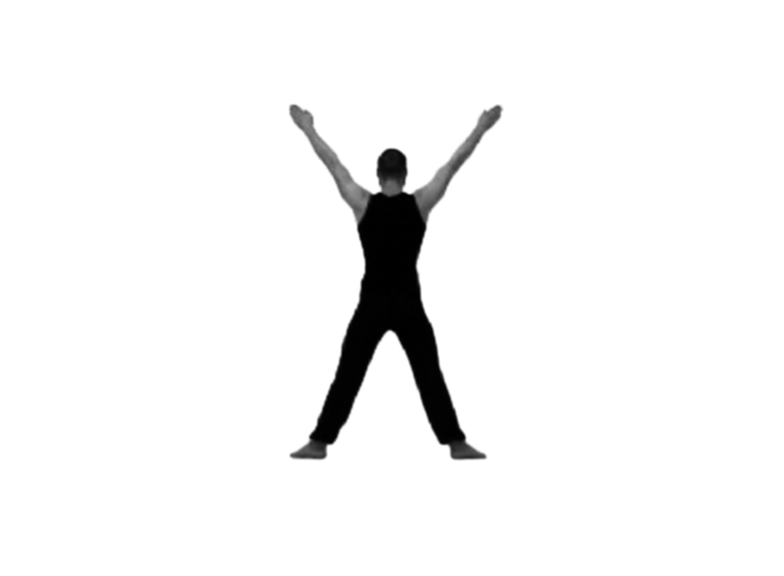

Supplement: Supplementary file 3 [file Data_Sheet_2.ZIP › Experiment 2 images/Postures/middle 3.png]

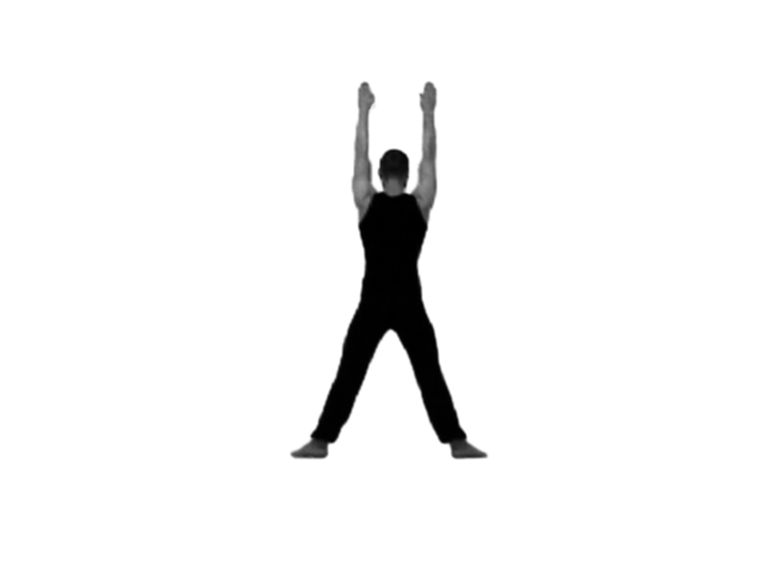

Supplement: Supplementary file 3 [file Data_Sheet_2.ZIP › Experiment 2 images/Postures/middle 4.png]

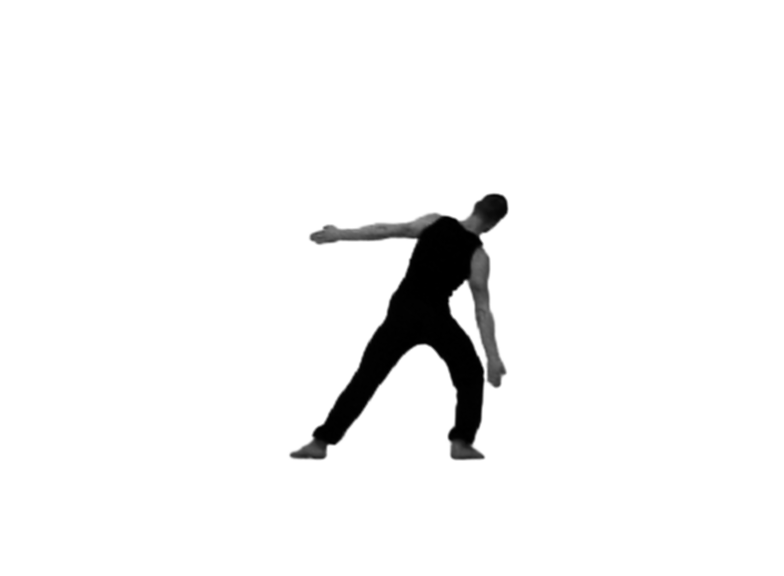

Supplement: Supplementary file 3 [file Data_Sheet_2.ZIP › Experiment 2 images/Postures/right 1.png]

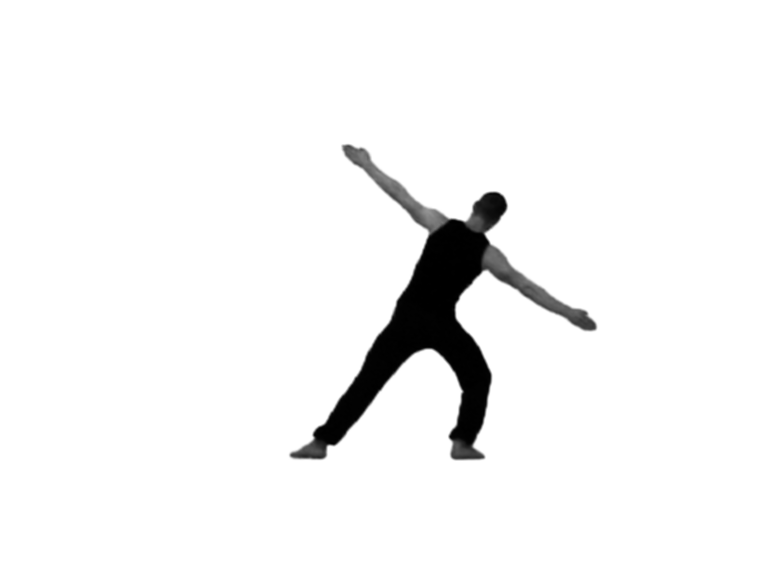

Supplement: Supplementary file 3 [file Data_Sheet_2.ZIP › Experiment 2 images/Postures/right 2.png]

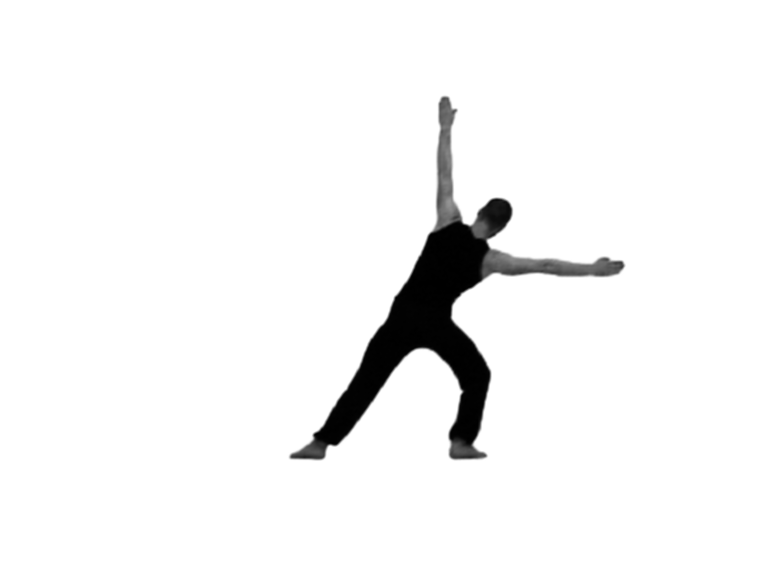

Supplement: Supplementary file 3 [file Data_Sheet_2.ZIP › Experiment 2 images/Postures/right 3.png]

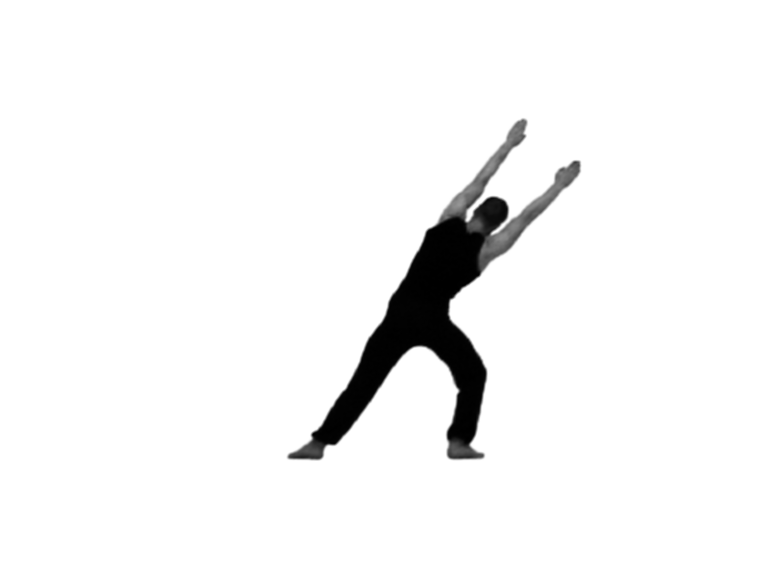

Supplement: Supplementary file 3 [file Data_Sheet_2.ZIP › Experiment 2 images/Postures/right 4.png]
